# Porous Organic Cage-Based Quasi-Solid-State Electrolyte with Cavity-Induced Anion-Trapping Effect for Long-Life Lithium Metal Batteries

**DOI:** 10.1007/s40820-024-01499-x

**Published:** 2024-10-15

**Authors:** Wei-Min Qin, Zhongliang Li, Wen-Xia Su, Jia-Min Hu, Hanqin Zou, Zhixuan Wu, Zhiqin Ruan, Yue-Peng Cai, Kang Li, Qifeng Zheng

**Affiliations:** 1 School of Chemistry, South China Normal University, Guangzhou, 510006 People’s Republic of China; 2 Key Laboratory of Functional Metal-Organic Compounds of Hunan Province, College of Chemistry and Material Science, Hengyang Normal University, Hengyang, 421008 People’s Republic of China; 3 Guangzhou Key Laboratory of Energy Conversion and Energy Storage Materials, Guangzhou, 510006 People’s Republic of China

**Keywords:** Porous organic cage, Cavity-induced anion-trapping, Quasi-solid-state electrolyte, Homogeneous Li^+^ flux, Lithium metal battery

## Abstract

Porous organic cages (POCs) with permanent porosity and excellent host–guest property hold great potentials in regulating ion transport behavior, yet their feasibility as solid-state electrolytes has never been testified in a practical battery. Herein, we design and fabricate a quasi-solid-state electrolyte (QSSE) based on a POC to enable the stable operation of Li-metal batteries (LMBs). Benefiting from the ordered channels and cavity-induced anion-trapping effect of POC, the resulting POC-based QSSE exhibits a high Li^+^ transference number of 0.67 and a high ionic conductivity of 1.25 × 10^−4^ S cm^−1^ with a low activation energy of 0.17 eV. These allow for homogeneous Li deposition and highly reversible Li plating/stripping for over 2000 h. As a proof of concept, the LMB assembled with POC-based QSSE demonstrates extremely stable cycling performance with 85% capacity retention after 1000 cycles. Therefore, our work demonstrates the practical applicability of POC as SSEs for LMBs and could be extended to other energy-storage systems, such as Na and K batteries.

**Supplementary Information:**

The online version contains supplementary material available at 10.1007/s40820-024-01499-x.

## Highlights

- A porous organic cage (POC)-based quasi-solid-state electrolyte (QSSE) with cavity-induced anion-trapping effect was rationally designed to enable the stable operation of Li-metal batteries.
- The POC-based QSSE exhibits a high Li^+^ transference number of 0.67 and a high ionic conductivity of 1.25×10^−4^ S cm^−1^ with a low activation energy of 0.17 eV.
- The POC-based QSSE demonstrates a highly reversible Li plating/stripping cycling for 2000 h and superior Li||LFePO_4_ cycling for thousands of cycles at room temperature.

**Supplementary Information:**

The online version contains supplementary material available at 10.1007/s40820-024-01499-x.

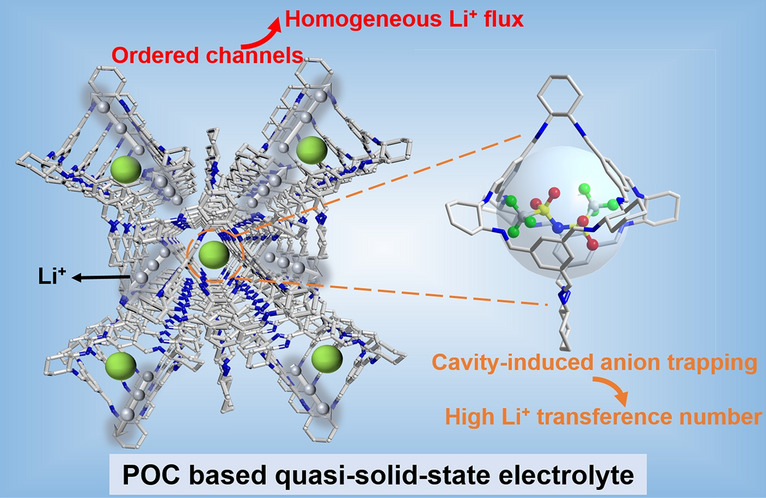

## Introduction

With the rapid development of portable electronic devices, electric vehicles, and energy storage grids, the demand for high energy density batteries is ever-increasing [1–3]. Lithium metal has been considered as the “Holy Grail” anode material for the next-generation batteries due to its lowest electrode potential (− 3.04 V vs. standard hydrogen electrode), small mass density (0.53 g cm^−3^), and highest theoretical specific capacity (3860 mAh g^−1^) [4–7]. However, traditional liquid electrolytes (LEs) with flammable and volatile properties are prone to form unstable solid electrolyte interphase (SEI) during cycling due to their high reactivity with lithium metal, resulting in uneven deposition of lithium and severe dendrite growth that can penetrate the separator to cause serious safety issues [8, 9].

Solid-state electrolytes (SSEs) are expected to become an ideal candidate for LMBs due to their nonflammable nature, low reactivity with lithium metal, and high mechanical strength that can suppress dendrite growth [10–13]. Although significant progress has been made in the SSEs field, their wide application is still limited by the poor interfacial contact between electrodes and electrolytes, as well as low ionic conductivity [14, 15], alternatively, with the introduction of a small amount of liquid to SSEs, namely quasi-solid-state electrolytes (QSSEs), whom not only maintain a solid-state phase to avoid leakage but also improve the interfacial contact between electrodes and electrolytes [16, 17]. Specifically, the liquid can act as a plasticizer to dissolve lithium salts and improve the ionic conductivity [18]. Recently, the mechanistic study on Li^+^ transportation also suggested that solvent-assisted Li^+^ hopping is the main transport pathway in QSSEs [19].

Porous organic cages (POCs), as an emerging class of crystalline molecular-based materials with permanent porosity [20], have found wide applications such as molecular recognition [21], separation [22–24], gas adsorption [25], and detection [26], with benefit from their porosity and excellent host–guest properties [27]. Different from other crystalline porous materials [28, 29], due to the discrete cage structure at the molecular level, the POCs can both dissolve in specific solvents and periodically stack to form crystalline organic solids, which would facilitate the solution-processing operation [30]. Meanwhile, POCs possess the following advantages in the solid state that bestows them great potential as SSEs: (1) crystalline nanoparticles with high surface areas are beneficial for improving the interfacial contact with electrodes; (2) the uniform porosity across the three-dimensional (3D) framework would facilitate the high-throughput transport with a homogeneous Li^+^ flux, leading to the uniform deposition of Li^+^ [31–36]. In 2015, Cooper et al. reported the first molecular cage with ionic transport capability, which showed excellent 3D protonic conductivity (~ 10^−3^ S cm^–1^) at relatively high humidity [37]. Gewirth et al. reported a Li^+^ conductor based on a tetrahedron POC (TD_A_) with bis(trifluoromethane)sulfonamide lithium salt (LiTFSI) in 1,2-dimethoxyethane (DME), which exhibited a high ionic conductivity of 1 × 10^−3^ S cm^−1^ [38]. Chen et al. prepared a Li^+^ conducting molecular cage (Li-RCC1-ClO_4_) as catholyte, which showed excellent solution processibility and dispersed uniformly in the slurry-coated cathodes to form a highly effective ion-conducting network, significantly improving the battery performance at room temperature [39]. Nevertheless, the feasibility of POC materials as SSEs for practical batteries has never been testified in previous publication.

**Fig. 1:**
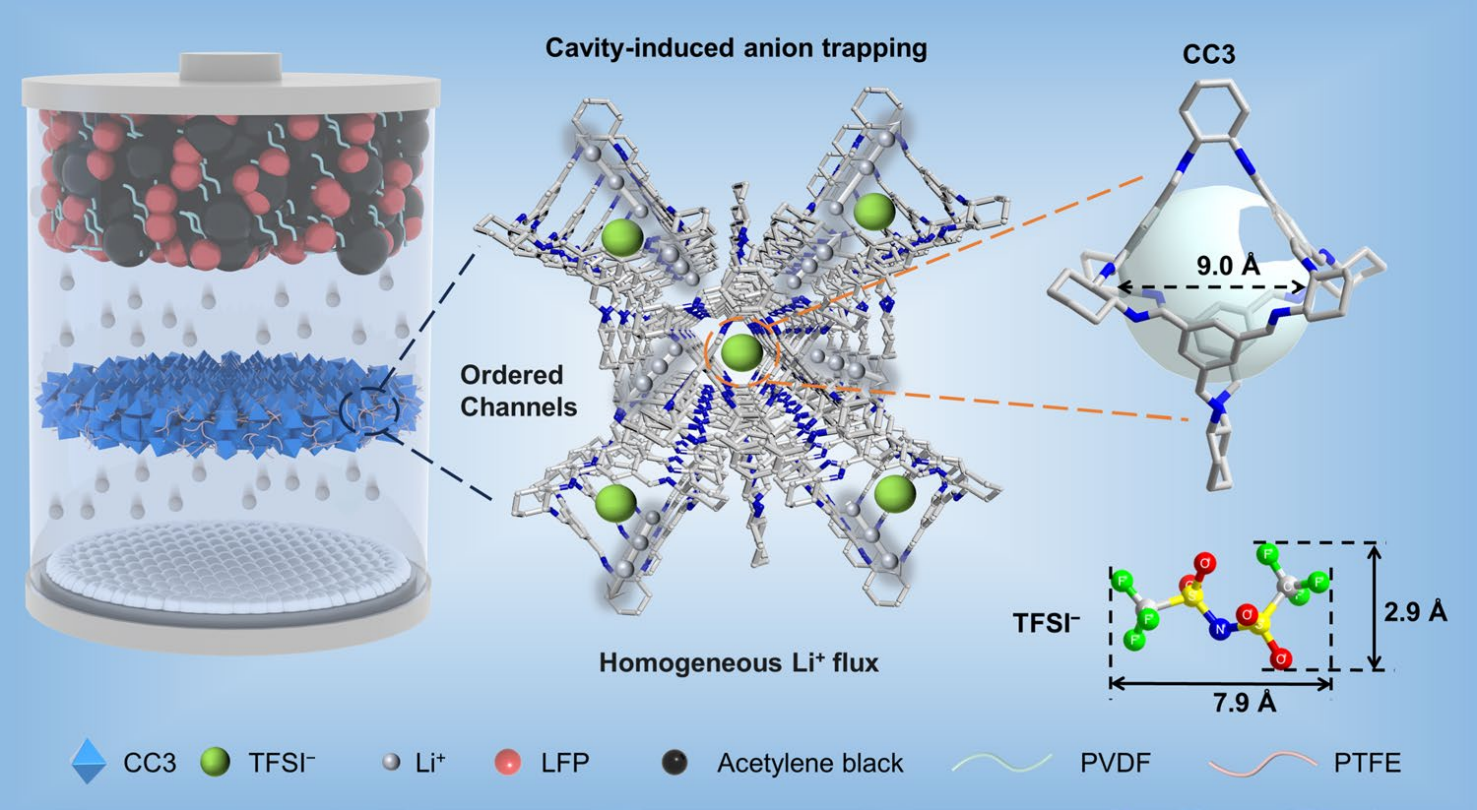
Schematic illustration of POC-based quasi-solid-state electrolyte (QSSE). The CC3 confines anions with cavity, and the Li^+^ transports along ordered channels of CC3 are expected to bestow the resulting QSSE with high Li^+^ transference number, high ionic conductivity, and homogeneous Li^+^ flux, leading to long-cycling dendrite-free LMBs

Considering POC’s 3D interconnected channels for efficient Li^+^ transport, as well as their cavities and pore size to restrict anions transport, herein, we designed a QSSE based on a reported POC [20] (i.e., CC3, C_72_H_84_N_12_, condensation between diaminocyclohexane and 1,3,5-benzenetricarboxaldehyde). As illustrated in Fig. 1, CC3 possesses an appropriate cavity size of 9.0 Å, which could serve as an effective anionic sieve to trap the TFSI^−^ anion (7.9 Å) and is supposed to weaken the electrostatic interactions between Li^+^–TFSI^−^ ion pairs, thereby significantly increasing the Li^+^ transport efficiency. Furthermore, the ordered channels formed through the discrete CC3 packing in crystal lattice would provide a multi-passway for Li^+^ high-throughput transport and homogeneous deposition. We envisioned that the assembled CC3-based QSSE would exhibit a high ionic conductivity and Li^+^ transference number with desired cycling stability for quasi-solid-state Li-metal batteries (LMBs) at room temperature.

## Experimental Section

### Synthesis of the CC3

CC3 was synthesized following a similar procedure to previous reports [40]. 1,3,5-Triformylbenzene (250 mg, 1.543 mmol) was dissolved in dichloromethane (5 mL) with the aid of sonication, and trifluoroacetic acid (5 μL) was then added. Thereafter, a solution of (R, R)-1,2-diaminocyclohexane (250 mg, 2.232 mmol) in dichloromethane (5 mL) was added carefully. After reacting for 5 days at room temperature, transparent prism-like crystals were formed. The solvent was removed by filtration, and a fresh mixed solution of dichloromethane (19 mL) and methanol (1 mL) was added to wash the crystals. Crystalline CC3 powders were produced after removing solvent by centrifugation and further vacuum-dried at 80 °C for 12 h, resulting in pure CC3 powder in a high yield (90%).

### Preparation of the CC3 Films and PTFE Films

In a mortar, CC3 and polytetrafluoroethylene (PTFE) emulsion were weighed at a mass ratio of 90:10. To aid in dispersion, several drops of ethanol were added. The films were created by combining emulsion and powder in a mortar and then running the mixture through a roller press multiple times. The films were divided into 16-mm-diameter circular plates and vacuum-dried for 12 h at 150 °C. To be used later, the plates were moved into the glove box. The preparation of PTFE films was similar to the CC3 films.

### Preparation of the CC3-Based QSSE

1.0 M LiTFSI in PC was used as liquid electrolyte (LE). To prepare the CC3-based QSSE, the CC3 films were immersed in the LE at 60 °C for 24 h and then wiped with dust-free paper and vacuum-dried for 10 h to remove the surface solvent.

### Preparation of the Cathode

The LiFePO_4_ (LFP) and LiCoO_2_ (LCO) cathodes were fabricated by mixing LFP/LCO powder, PVDF, and acetylene black at a mass ratio of 80: 10: 10 in NMP using a mixer (AR-100, Thinky). The as-prepared slurry was subsequently coated on aluminum foil and dried under vacuum at 120 °C for 24 h. The mass loading of the active materials was at ~ 3 mg cm^−2^.

## Results and Discussion

### Design Rationale and Fabrication of the POC-Based QSSE

**Fig. 2:**
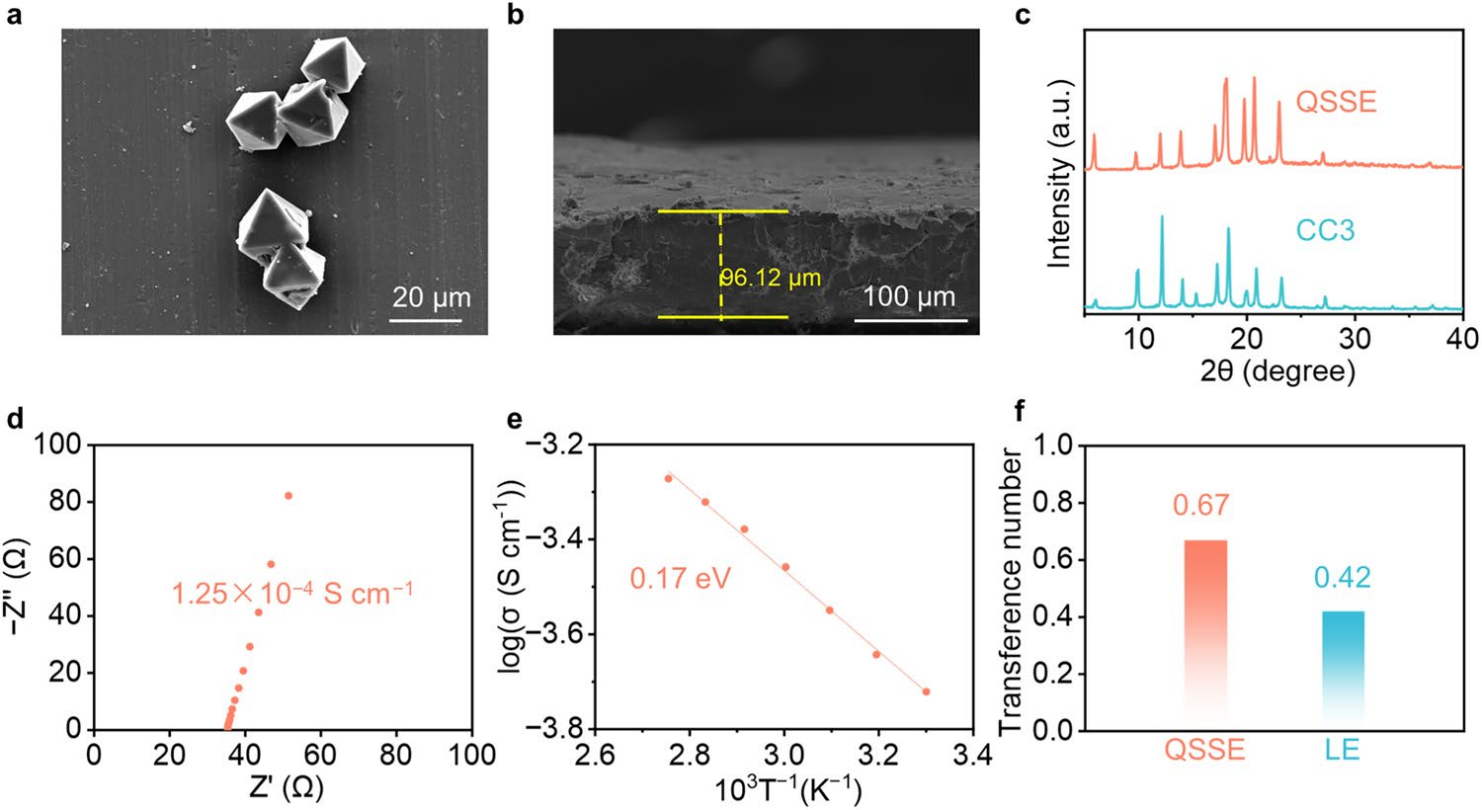
Characterizations of the QSSE. SEM images of **a** CC3 crystals and **b** cross-sectional view of QSSE. **c** Powder X-ray diffraction patterns of CC3 and QSSE. **d** Ionic conductivity of QSSE at 25 °C. **e** Temperature-dependent ionic conductivities of QSSE. **f** Transference number of QSSE and LE

The molecular CC3 was synthesized by the direct condensation between 1,3,5-triformylbenzene and 1,2-diaminocyclohexane (Fig. S1) as reported by the literature [40] and identified by nuclear magnetic resonance (NMR) spectroscopy (Figs. S2 and S3). After evaporation, the crystalline CC3 solid was obtained with a high yield (90%) and its phase purity was verified by powder X-ray diffraction (PXRD) method (Fig. S4). As shown in Fig. 2a, the regular and uniform octahedron-shaped crystals on a micrometer scale were observed by scanning electron microscope (SEM), indicating the excellent crystallinity for CC3 solid. Subsequently, the CC3 was grounded with polytetrafluoroethylene (PTFE) to prepare a homogeneous and flexible film through rolling. For comparison purpose, the pure PTFE film (i.e., without CC3) was also prepared following a similar process. As shown in Fig. S5, the size of CC3 in the film is at the nanoscale, and the uniform distribution can be observed from energy-dispersive spectroscopy (EDS). After that, the film was immersed in the solution of 1.0 M LiTFSI in PC at 60 °C for 1 day to prepare the QSSE. To investigate the liquid content in the QSSE, thermogravimetric (TG) testing was carried out for both CC3-based QSSE and pure PTFE film after soaking with the liquid electrolyte. As shown in Fig. S6, it is of about 12% (mass fraction) PC solvent existed in the CC3-based QSSE, while PTFE film did not show significant mass change, indicating that it could not adsorb liquid electrolyte at all. The content of LiTFSI in QSSE was determined to be 11.4 wt% according to the inductively coupled plasma optical emission spectrometry (ICP-OES). The thickness of the QSSE film was measured to be approximately 96 μm from the SEM image (Fig. 2b). Furthermore, according to the PXRD results, it was found that the characteristic diffraction peaks of the QSSE match well with that of CC3, suggesting the CC3 in QSSE still maintains its original structure (Fig. 2c). To quantify and compare the porosity of two materials, the CC3 and fabricated QSSE were subjected to nitrogen adsorption and desorption measurement, respectively. The results showed that CC3 has a high specific surface area of 426 m^2^ g^−1^, while QSSE holds a relatively low specific surface area of 7 m^2^ g^−1^ (Fig. S7). This implied that the introduced electrolyte in QSSE may enter and occupy the pores of the CC3 framework, thus causing a substantial decrease in the adsorption capacity and specific surface area.

With a small amount of PC for plasticization, the QSSE film showed a high ionic conductivity of 1.25 × 10^−4^ S cm^−1^ (Fig. 2d). On the contrary, since the PTFE film is incapable to adsorb liquid electrolyte at all, it only exhibited an extremely low ionic conductivity of 9.45 × 10^−8^ S cm^−1^ (Fig. S8). Consequently, the ionic conductivity at different temperatures was screened, and the active energy of QSSE was calculated to be 0.17 eV (Fig. 2e) according to Arrhenius equation, indicating that the Li^+^ transport is less affected by the temperature parameter and low energy barriers are needed for Li^+^ transport in 3D interconnected channels of CC3. This phenomenon was attributed to the solvent-assisted Li^+^ hopping conduction in CC3-based QSSE with low Li^+^ migration barrier, which has been demonstrated in other crystalline porous materials-based QSSEs [19]. Specifically, as illustrated in Fig. S9, within the hierarchical pores formed by the stacking of CC3, Li^+^ coordinates with PC and the most electronegative N atom on CC3. When Li^+^ jump, they will dissociate from the coordinated N atoms, followed by total solvation with PC. Thereafter, the Li^+^ will jump to the adjacent CC3 to coordinate with the N atoms, with the excess PC dissociated from coordination.

Linear sweep voltammetry (LSV) was used to assess the electrochemical windows of the QSSE and LE (i.e., 1.0 M LiTFSI in PC). The result demonstrated that the oxidation potential of LE was 4.3 V, while an oxidation potential up to 4.7 V was achieved for QSSE (Fig. S10). This may be due to the confinement effect of CC3 that reduces the free solvent (i.e., PC) in QSSE, which extends the electrochemical window. The Li^+^ transference number plays an important role in enabling the stable operation of LMBs, where high Li^+^ transference number indicates high Li^+^ mobility, and it is beneficial for homogenizing Li^+^ flux and suppressing the growth of Li dendrites [41]. As shown in Figs. 2f and S11, the QSSE film showed a higher Li^+^ transference number of 0.67 than the corresponding LE counterpart (0.42).

### Cavity-Induced Anion-Trapping Effect

**Fig. 3:**
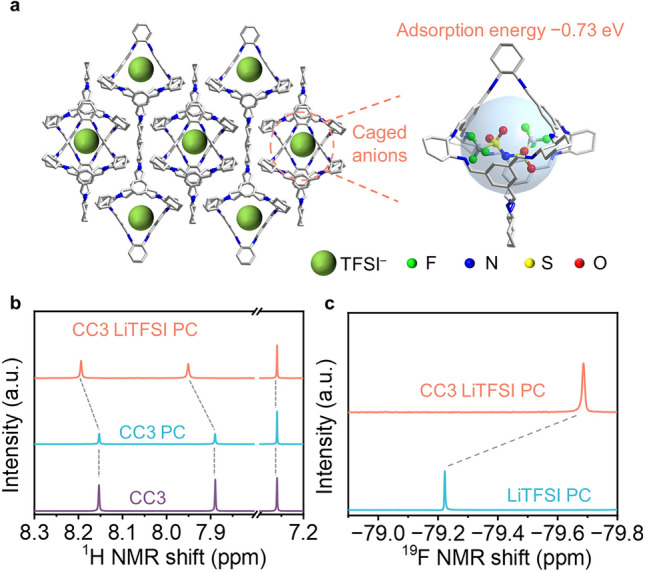
Investigation of the interaction between TFSI^−^ and CC3. **a** Packing structure of CC3 and the adsorption energy of TFSI^−^ in the cavity. **b** ^1^H NMR of CC3, CC3 with PC, and CC3 with LiTFSI in PC in CDCl_3_. **c**^19^F NMR of LiTFSI in PC and LiTFSI in PC with CC3 in CDCl_3_

To disclose the origin of the high Li^+^ transference number in QSSE, the interaction between the TFSI^−^ and CC3 cavity was evaluated by calculating the adsorption energy of TFSI^−^ in the cavity of CC3. As shown in Figs. S12 and S13, the CC3 has an adsorption energy of − 0.73 eV with TFSI^−^, which is much higher than the recently reported Li^+^ conductor based on a tetrahedron POC (i.e., TD_A_) with an adsorption energy of − 0.59 eV. This difference may be ascribed to the proximity of the cavity size of CC3 (9.0 Å) to TFSI^−^ anion (7.9 Å), while the cavity size of TD_A_ (14 Å) is much larger than the anions [42], implying that TFSI^−^ could be tightly restricted in the cavity of CC3 in an electric field (Fig. 3a). In other words, the larger proportion of Li^+^ would move rapidly in CC3-based QSSE, thus resulting in a relatively high Li^+^ transference number. Therefore, a prolonged initial time of Li dendrite growth in QSSE could be anticipated, demonstrating the superiority of CC3-based QSSE.

Meanwhile, to gain more information about the interaction between CC3 and TFSI^−^ anions, the NMR spectra were recorded for investigating the effect of CC3 on the TFSI^−^. Firstly, when adding PC to the CC3 solution, the two characteristic chemical shifts (7.91 and 8.18 ppm) assigned to CC3 protons were almost unchanged, indicating that the PC molecule has little interaction with CC3 (Fig. 3b). Next, when LiTFSI was introduced, the two peaks of CC3 showed a distinct shift to downfield (0.1 ppm), which could be attributed to the van der Waals force between TFSI^−^ and CC3. The TFSI^−^ anion containing strong electron-withdrawing atoms (F, O) would reduce the electron density of CC3 skeleton once immobilizing by the cage cavities [43]. Furthermore, in ^19^F NMR spectra, the chemical shift of TFSI^−^ anion with the presence of CC3 significantly moved to the upfield (0.4 ppm) compared with the individual LiTFSI (Fig. 3c). This prominent shift is attributed to the confinement effect of the CC3 cage, which can shield the electromagnetic field of accommodated anion in the cavity by its exterior aromatic panels. This phenomenon is commonly found in the cage’s host–guest interactions field [44]. Together, both ^1^H and ^19^F NMR showed the noteworthy interactions between CC3 and TFSI^−^, demonstrating that CC3 has a significant cavity-induced anion-trapping effect on TFSI^−^.

### Superior Compatibility Toward Li-Metal

To investigate the compatibility between the QSSE film and Li-metal anode, the Li||Li symmetric cells were assembled, and the critical current density (CCD) was first investigated. As shown in Fig. S14, the CCD of the cell with LE is 1.25 mA cm^−2^, while the cell using QSSE reaches 1.60 mA cm^−2^, indicating that QSSE possesses a potential ability to suppress the growth of dendrite at high current density. The superior compatibility was further verified by repeated Li plating/stripping cycling test. Under the condition of 0.1 mA cm^−2^ for 0.1 mAh cm^−2^, the Li||QSSE||Li cell achieved an extremely stable cycling for over 2000 h, with a stable polarization voltage at around 30 mV. In sharp contrast, the polarization voltage of the Li||LE||Li began to increase significantly after 700 h and became as high as of 100 mV at approximately 1000 h (Fig. S15). The interfacial evolution of the Li||Li cells during the cycling process was evaluated by testing the impedance at different cycling times. As shown in Fig. S16, the interfacial resistance of the Li||Li cell using QSSE maintains very low and stable during cycling, while that of using LE increases sharply after 700 h, which is consistent with the significant increase in polarization voltage during the cycling process.

**Fig. 4:**
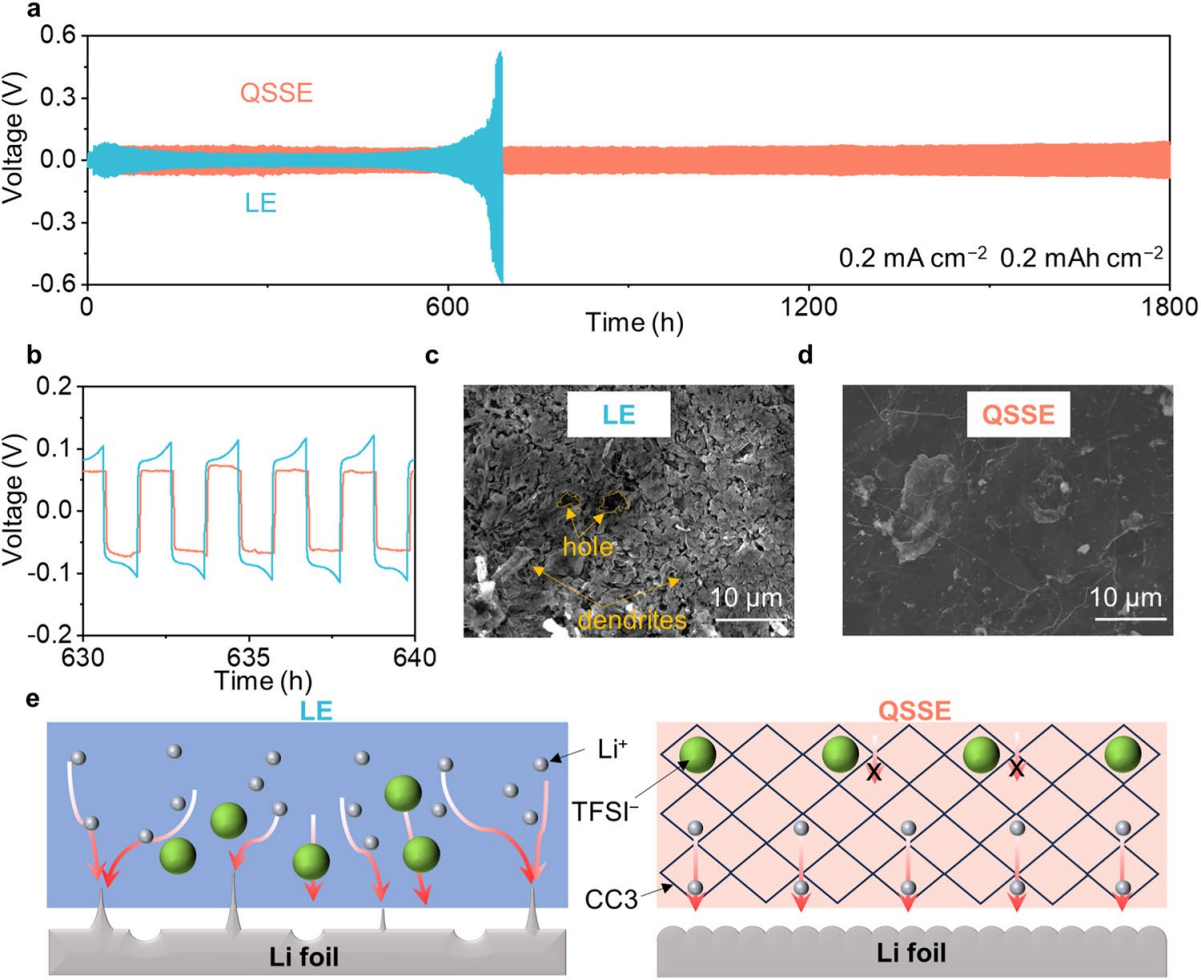
Li-metal compatibility in QSSE. **a** Long-term cycling of Li||Li symmetric cells at 0.2 mA cm^−2^ for 0.2 mAh cm^−2^. **b** Enlarged voltage profiles. SEM images of Li-metal electrode using **c** LE and **d** QSSE after 50 cycles at 0.2 mA cm^−2^ for 0.2 mAh cm^−2^. **e** Illustration of Li deposition behaviors of cells with LE and QSSE (compared to the LE, in QSSE, CC3 with ordered channels and suitable cavity was designed to restrict the movement of TFSI^−^ anions, leading to homogeneous Li^+^ flux, high t_Li_^+^, and dendrite-free morphology)

Moreover, at a higher current density of 0.2 mA cm^−2^ for 0.2 mAh cm^−2^, the symmetric cells with QSSE could stably cycle for over 1800 h, while the polarization voltage of the cell with LE increased significantly after 600 h and eventually sudden short-circuited at 700 h (Fig. 4a, b). The Li-metal surface morphology after cycling was studied by SEM. Obvious Li dendrites and porous deposition morphology were observed on the Li-metal surface in Li||LE||Li cell (Fig. 4c), which would increase the interfacial resistance and puncture the separator to cause serious short circuit. In contrast, the Li-metal surface is quite smooth and uniform for Li||QSSE||Li, with no obvious dendrites observed (Fig. 4d). When the current density was further increased to 0.5 mA cm^−2^, the symmetry cell with LE experienced severe voltage fluctuations and short-circuited within only 50 h, while the Li||QSSE||Li operated stably for over 250 h (Fig. S17). Even at 1.0 mA cm^−2^, batteries with QSSE can operate stably for more than 100 h and the polarization voltage essentially stays constant (Fig. S18). After the cycling of Li||Li cells, the morphology and flexibility of QSSE were almost the same as before cycling, and the crystalline structure of QSSE was ensured to be retained with no significant damage, which is important for the prolong cycling of LMBs (Figs. S19 and S20). Together, the Li deposition behavior is illustrated in Fig. 4e; with the aid of cavity-induced anion-trapping effect of CC3, the movement of the TFSI^−^ anion was effectively restricted to promote the free transport of Li^+^, resulting in a high Li^+^ transference number that minimizes the concentration gradient during cycling, which postpones the initial growth time of Li dendrites. In addition, 3D periodic and interconnected channels of CC3 can homogenize the Li^+^ flux that leads to the homogeneous deposition of Li^+^.

**Fig. 5:**
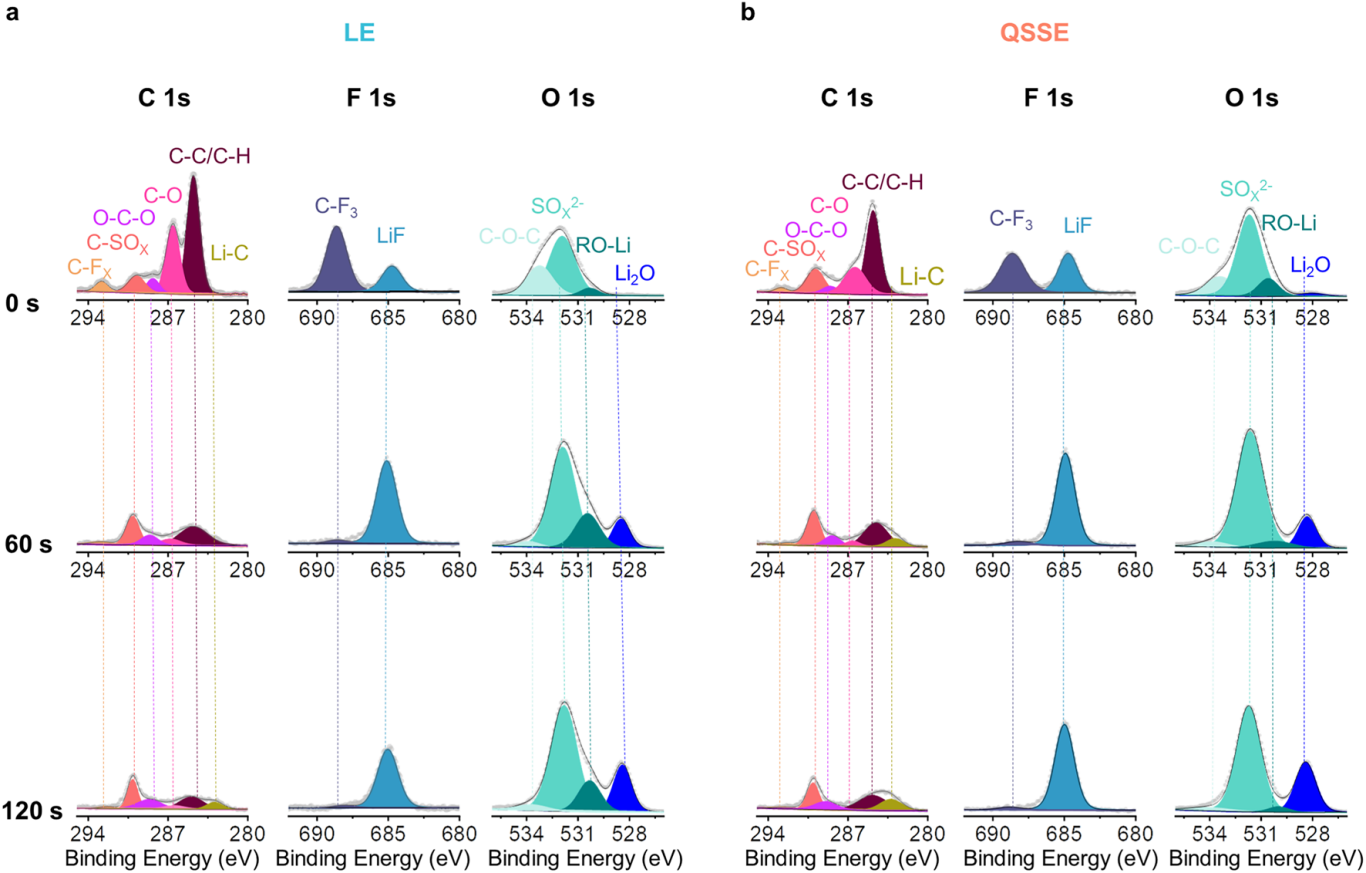
Characterization of the SEI on the Li electrode. XPS depth profiles of the Li-metal electrode in **a** LE and **b** QSSE at 0.2 mA cm^−2^ with 0.2 mAh cm^−2^ after 50 plating/stripping cycles

The SEI chemistry is crucial regulating the Li deposition behavior; therefore, the X-ray photoelectron spectroscopy (XPS) with an Ar^+^ sputtering technique was adopted on the Li-metal anode after plating/stripping for 50 cycles. As shown in Fig. 5a, for LE system, a main distribution of organic components (mainly as lithium alkoxy) considered to be unstable and resistive was observed on the surface of Li-metal, which was derived from the PC solvent. Simultaneously, organic fluoride component (i.e., CF_3_) with a small amount of LiF component was also observed, which was derived from the incomplete decomposition of TFSI^−^ anions, while for the Li-metal in QSSE, except for a small portion of organic components, an increased ratio of fluorine-containing species, especially for the inorganic species (i.e., LiF), was found. After Ar^+^ sputtering, the inorganic components were further enhanced, where particularly strong signals of LiF and sulfur-containing species could still be observed even after 120 s of sputtering. These results indicate that, compared to that of using LE, more inorganic SEI components were generated for the cell using QSSE, which suggests that more anions were preferentially reduced to form inorganic-rich SEI with low resistance that improves the interfacial stability and enhances the reversibility of Li plating/stripping. This may be attributed to the solvent sieving effect of POC that promotes the formation of more contact ion pairs (CIPs) according to the Raman study (Fig. S21), of which the similar phenomenon has been demonstrated in previous publications [45, 46].

### Stable Operation of the LMBs

**Fig. 6:**
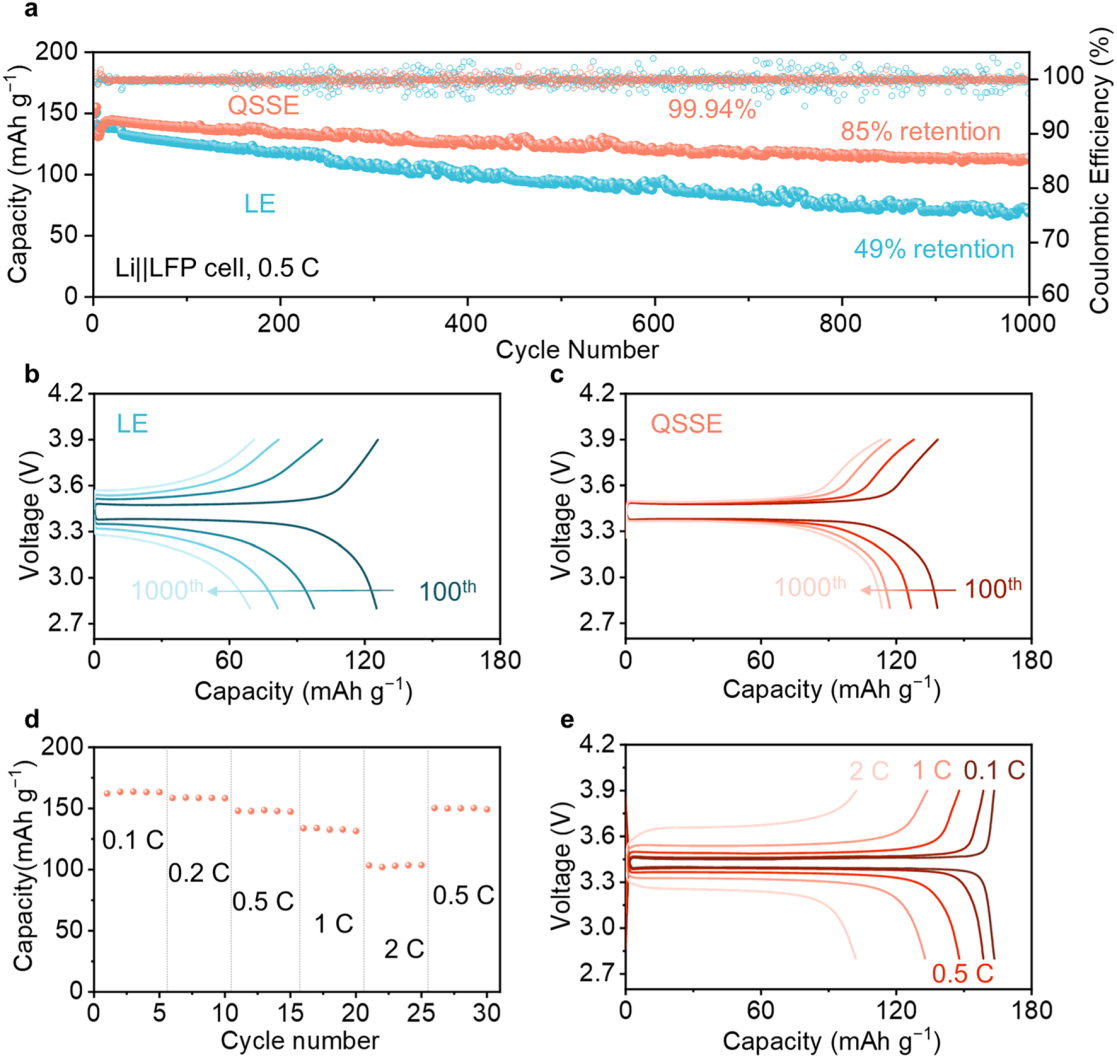
Electrochemical performance of Li||LFP cells at room temperature. **a** Long-term cycling of Li||LFP cells at 0.5 C after 3 formation cycles at 0.1 C. Charge–discharge curves at different cycles for Li||LFP cells using **b** LE and **c** QSSE. **d** Rate performance of Li|| LFP cell with QSSE. **e** Charge–discharge curves of Li||LFP cell with QSSE at different rates

The superiority of the as-fabricated QSSE was eventually validated in the Li||LiFePO_4_(LFP) full cells. As shown in the long cycling test of cells at 0.5 C at room temperature (Fig. 6a), the cell using QSSE still maintained a high capacity of 113.7 mAh g^−1^ after 1000 cycles, corresponding to a capacity retention rate of 85%. In comparison, in the LE system, the specific capacity was only 69.1 mAh g^−1^ with a low capacity retention of only 49% after 1000 cycles. Meanwhile, the Coulombic efficiency (CE) fluctuated violently for the cell using LE, indicating that the interfaces between the electrodes and LE are unstable during cycling. In contrast, the cell using QSSE system achieved a stable and ultra-high average CE of 99.94% without fluctuation, demonstrating a much more stable interface between the electrodes and QSSE. The charging–discharging curves at different cycles (100th, 400th, 700th, and 1000th) were presented (Fig. 6b, c). The cell using QSSE exhibited low polarization voltage with no obvious increase during cycling, indicating excellent interfacial compatibility of the QSSE toward both LFP cathode and Li-metal anode. However, the cell using LE exhibited significant capacity decay and distinct polarization during cycling, which is mainly ascribed to the uneven deposition of Li and accumulation of dead Li. After the cycling, the cells were disassembled to visualize the morphology of the Li-metal surface (Fig. S22). The cell with LE showed an extremely uneven, loose, and inflated surface with sharp lithium dendrite existed, while the surface of Li-metal in QSSE is much flat, dense, and uniform without lithium dendrite. Furthermore, the rate performance of the cell using QSSE was assessed (Fig. 6d, e), which gave a specific capacity of 163, 158, 148, 132, and 103 mAh g^−1^ at 0.1, 0.2, 0.5, 1, and 2 C, respectively, demonstrating its potential for practical application. In addition, benefiting from the superiority oxidation stability (Fig. S10), the QSSE also enables stable operation of high-voltage LiCoO_2_ (LCO) cathode under a high cutoff voltage of 4.3 V, as indicated by no capacity decay after 100 cycles at 1.0 C (Fig. S23). Such performance outperforms most of the previously reported QSSEs (Table S1).

## Conclusions

In summary, a new QSSE based on CC3 POC was rationally designed to regulate the ion transport behavior with cavity-induced anion-trapping effect to enable long-life Li-metal batteries (LMBs). The cavity of CC3 could confine the movements of anions to promote the free transport of Li^+^, thus affording a high Li^+^ transference number of 0.67. The ordered channels packing in CC3 could homogenize the Li^+^ flux with high throughput, delivering a high ionic conductivity of 1.25 × 10^−4^ S cm^−1^. Furthermore, the solvent sieving effect of CC3 also leads to the formation of a uniform inorganic-rich SEI. Benefiting from the above merits that could effectively suppress the growth of Li dendrites, the resulting CC3-based QSSE yields highly smooth Li deposition morphology and remarkably reversible Li plating/stripping cycling for over 2000 h. Moreover, the QSSE also allows the Li||LFP full cell to operate stably over 1000 cycles (85% capacity retained) with a high average CE of 99.94% at room temperature. Depending on this unique cage material and its prominent confinement effect, this work provides an alternative strategy to construct quasi-solid-state electrolytes for LMBs from the supramolecular perspective.

## Supplementary Information

Below is the link to the electronic supplementary material.

Supplementary file1 (DOCX 4301 kb)

## References

[1] A. Li, X. Liao, H. Zhang, L. Shi, P. Wang et al., Nacre-inspired composite electrolytes for load-bearing solid-state lithium-metal batteries. Adv. Mater. **32**, e1905517 (2020). 10.1002/adma.201905517 10.1002/adma.201905517 31782563

[2] C.Y. Wang, T. Liu, X.G. Yang, S. Ge, N.V. Stanley et al., Fast charging of energy-dense lithium-ion batteries. Nature **611**, 485–490 (2022). 10.1038/s41586-022-05281-0 10.1038/s41586-022-05281-0 36224388

[3] J. Liu, Y. Zhang, J. Zhou, Z. Wang, P. Zhu et al., Advances and prospects in improving the utilization efficiency of lithium for high energy density lithium batteries. Adv. Funct. Mater. **33**, 2302055 (2023). 10.1002/adfm.202302055

[4] B. Acebedo, M.C. Morant, E. Gonzalo, I. Ruiz de Larramendi, A. Villaverde et al., Current status and future perspective on lithium metal anode production methods. Adv. Energy Mater. **13**, 2203744 (2023). 10.1002/aenm.202203744

[5] W. Xu, J. Wang, F. Ding, X. Chen, E. Nasybulin et al., Lithium metal anodes for rechargeable batteries. Energy Environ. Sci. **7**, 513–537 (2014). 10.1039/c3ee40795k

[6] P. Bonnick, J. Muldoon, The quest for the holy grail of solid-state lithium batteries. Energy Environ. Sci. **15**, 1840–1860 (2022). 10.1039/d2ee00842d

[7] D. Lin, Y. Liu, Y. Cui, Reviving the lithium metal anode for high-energy batteries. Nat. Nanotech. **12**, 194–206 (2017). 10.1038/nnano.2017.16 10.1038/nnano.2017.16 28265117

[8] R. Chen, Q. Li, X. Yu, L. Chen, H. Li, Approaching practically accessible solid-state batteries: stability issues related to solid electrolytes and interfaces. Chem. Rev. **120**, 6820–6877 (2020). 10.1021/acs.chemrev.9b00268 10.1021/acs.chemrev.9b00268 31763824

[9] L. Qian, T. Or, Y. Zheng, M. Li, D. Karim et al., Critical operation strategies toward high-performance lithium metal batteries. Renewables **1**, 114–141 (2023). 10.31635/renewables.023.202200014

[10] M.J. Wang, E. Kazyak, N.P. Dasgupta, J. Sakamoto, Transitioning solid-state batteries from lab to market: linking electro-chemo-mechanics with practical considerations. Joule **5**, 1371–1390 (2021). 10.1016/j.joule.2021.04.001

[11] T. Zhang, W. He, W. Zhang, T. Wang, P. Li et al., Designing composite solid-state electrolytes for high performance lithium ion or lithium metal batteries. Chem. Sci. **11**, 8686–8707 (2020). 10.1039/d0sc03121f 10.1039/d0sc03121f PMC8162172 34094187

[12] J. Lee, T. Lee, K. Char, K.J. Kim, J.W. Choi, Issues and advances in scaling up sulfide-based all-solid-state batteries. Acc. Chem. Res. **54**, 3390–3402 (2021). 10.1021/acs.accounts.1c00333 10.1021/acs.accounts.1c00333 34402619

[13] C. Liao, C. Yu, S. Chen, C. Wei, Z. Wu et al., Mitigation of the instability of ultrafast li-ion conductor Li_6.6_Si_0.6_Sb_0.4_S_5_I enables high-performance all-solid-state batteries. Renewables **1**, 266–276 (2023). 10.31635/renewables.023.202200021

[14] M. Dirican, C. Yan, P. Zhu, X. Zhang, Composite solid electrolytes for all-solid-state lithium batteries. Mater. Sci. Eng., R **136**, 27–46 (2019). 10.1016/j.mser.2018.10.004

[15] S. Xia, X. Wu, Z. Zhang, Y. Cui, W. Liu, Practical challenges and future perspectives of all-solid-state lithium-metal batteries. Chem **5**, 753–785 (2019). 10.1016/j.chempr.2018.11.013

[16] Z. Liu, W. Chen, F. Zhang, F. Wu, R. Chen et al., Hollow-particles quasi-solid-state electrolytes with biomimetic ion channels for high-performance lithium-metal batteries. Small **19**, e2206655 (2023). 10.1002/smll.202206655 10.1002/smll.202206655 36737835

[17] Q. Zhang, B. Liu, J. Wang, Q. Li, D. Li et al., The optimized interfacial compatibility of metal-organic frameworks enables a high-performance quasi-solid metal battery. ACS Energy Lett. **5**, 2919–2926 (2020). 10.1021/acsenergylett.0c01517

[18] J. Zhou, H. Ji, J. Liu, T. Qian, C. Yan, A new high ionic conductive gel polymer electrolyte enables highly stable quasi-solid-state lithium sulfur battery. Energy Storage Mater. **22**, 256–264 (2019). 10.1016/j.ensm.2019.01.024

[19] T. Hou, W. Xu, X. Pei, L. Jiang, O.M. Yaghi et al., Ionic conduction mechanism and design of metal-organic framework based quasi-solid-state electrolytes. J. Am. Chem. Soc. **144**, 13446–13450 (2022). 10.1021/jacs.2c03710 10.1021/jacs.2c03710 PMC9377385 35700972

[20] T. Tozawa, J.T. Jones, S.I. Swamy, S. Jiang, D.J. Adams et al., Porous organic cages. Nat. Mater. **8**, 973–978 (2009). 10.1038/nmat2545 10.1038/nmat2545 19855385

[21] D.X. Cui, Y. Geng, J.N. Kou, G.G. Shan, C.Y. Sun et al., Chiral self-sorting and guest recognition of porous aromatic cages. Nat. Commun. **13**, 4011 (2022). 10.1038/s41467-022-31785-4 10.1038/s41467-022-31785-4 PMC9273608 35817768

[22] A. He, Z. Jiang, Y. Wu, H. Hussain, J. Rawle et al., A smart and responsive crystalline porous organic cage membrane with switchable pore apertures for graded molecular sieving. Nat. Mater. **21**, 463–470 (2022). 10.1038/s41563-021-01168-z 10.1038/s41563-021-01168-z PMC8971131 35013552

[23] Q. Zhang, H. Li, S. Chen, J. Duan, W. Jin, Mixed-matrix membranes with soluble porous organic molecular cage for highly efficient C_3_H_6_/C_3_H_8_ separation. J. Membr. Sci. **611**, 118288 (2020). 10.1016/j.memsci.2020.118288

[24] T. Xu, B. Wu, L. Hou, Y. Zhu, F. Sheng et al., Highly ion-permselective porous organic cage membranes with hierarchical channels. J. Am. Chem. Soc. **144**, 10220–10229 (2022). 10.1021/jacs.2c00318 10.1021/jacs.2c00318 35586909

[25] K. Tian, S.M. Elbert, X.Y. Hu, T. Kirschbaum, W.S. Zhang et al., Highly selective adsorption of perfluorinated greenhouse gases by porous organic cages. Adv. Mater. **34**, e2202290 (2022). 10.1002/adma.202202290 10.1002/adma.202202290 35657163

[26] P.E. Alexandre, W.S. Zhang, F. Rominger, S.M. Elbert, R.R. Schröder et al., A robust porous quinoline cage: transformation of a [4+6] salicylimine cage by povarov cyclization. Angew. Chem. Int. Ed. **59**, 19675–19679 (2020). 10.1002/anie.202007048 10.1002/anie.202007048 PMC7689861 32521080

[27] X. Yang, Z. Ullah, J.F. Stoddart, C.T. Yavuz, Porous organic cages. Chem. Rev. **123**, 4602–4634 (2023). 10.1021/acs.chemrev.2c00667 10.1021/acs.chemrev.2c00667 PMC10141292 37023354

[28] J.F. Zhang, Y.Y. Wang, X.F. Li, G.Y. Zhang, Y. Li et al., ZIF-8-functionalized polymer electrolyte with enhanced performance for high-temperature solid-state lithium metal batteries. Rare Met. **43**, 984–994 (2023). 10.1007/s12598-023-02521-8

[29] X. Guan, Z. Jian, X. Liao, W. Liao, Y. Huang et al., Tailored architecture of composite electrolyte for all-solid-state sodium batteries with superior rate performance and cycle life. Nano Res. **17**, 4171–4180 (2024). 10.1007/s12274-023-6354-y

[30] T. Hasell, A.I. Cooper, Porous organic cages: soluble, modular and molecular pores. Nat. Rev. Mater. **1**, 1–14 (2016). 10.1038/natrevmats.2016.53

[31] R. Zhao, Y. Wu, Z. Liang, L. Gao, W. Xia et al., Metal-organic frameworks for solid-state electrolytes. Energy Environ. Sci. **13**, 2386–2403 (2020). 10.1039/d0ee00153h

[32] G. Zhang, Y.L. Hong, Y. Nishiyama, S. Bai, S. Kitagawa et al., Accumulation of glassy poly(ethylene oxide) anchored in a covalent organic framework as a solid-state Li^+^ electrolyte. J. Am. Chem. Soc. **141**, 1227–1234 (2019). 10.1021/jacs.8b07670 10.1021/jacs.8b07670 30576136

[33] W. Gong, Y. Ouyang, S. Guo, Y. Xiao, Q. Zeng et al., Covalent organic framework with multi-cationic molecular chains for gate mechanism controlled superionic conduction in all-solid-state batteries. Angew. Chem. Int. Ed., e202302505 (2023). 10.1002/anie.202302505 10.1002/anie.202302505 36992624

[34] Y. An, S. Tan, Y. Liu, K. Zhu, L. Hu et al., Designs and applications of multi-functional covalent organic frameworks in rechargeable batteries. Energy Storage Mater. **41**, 354–379 (2021). 10.1016/j.ensm.2021.06.010

[35] Z. Wang, Z. Wang, L. Yang, H. Wang, Y. Song et al., Boosting interfacial Li^+^ transport with a MOF-based ionic conductor for solid-state batteries. Nano Energy **49**, 580–587 (2018). 10.1016/j.nanoen.2018.04.076

[36] C. Zhang, L. Shen, J. Shen, F. Liu, G. Chen et al., Anion-sorbent composite separators for high-rate lithium-ion batteries. Adv. Mater. **31**, e1808338 (2019). 10.1002/adma.201808338 10.1002/adma.201808338 30957302

[37] M. Liu, L. Chen, S. Lewis, S.Y. Chong, M.A. Little et al., Three-dimensional protonic conductivity in porous organic cage solids. Nat. Commun. **7**, 12750 (2016). 10.1038/ncomms12750 10.1038/ncomms12750 PMC5027280 27619230

[38] A. Petronico, B.G. Nicolau, J.S. Moore, R.G. Nuzzo, A.A. Gewirth, Solid-liquid lithium electrolyte nanocomposites derived from porous molecular cages. J. Am. Chem. Soc. **140**, 7504–7509 (2018). 10.1021/jacs.8b00886 10.1021/jacs.8b00886 29860840

[39] J. Li, J. Qi, F. Jin, F. Zhang, L. Zheng et al., Room temperature all-solid-state lithium batteries based on a soluble organic cage ionic conductor. Nat. Commun. **13**, 2031 (2022). 10.1038/s41467-022-29743-1 10.1038/s41467-022-29743-1 PMC9018795 35440112

[40] M. Liu, M.A. Little, K.E. Jelfs, J.T. Jones, M. Schmidtmann et al., Acid- and base-stable porous organic cages: shape persistence and pH stability via post-synthetic "tying" of a flexible amine cage. J. Am. Chem. Soc. **136**, 7583–7586 (2014). 10.1021/ja503223j 10.1021/ja503223j 24785267

[41] X.B. Cheng, R. Zhang, C.Z. Zhao, Q. Zhang, Toward safe lithium metal anode in rechargeable batteries: a review. Chem. Rev. **117**, 10403–10473 (2017). 10.1021/acs.chemrev.7b00115 10.1021/acs.chemrev.7b00115 28753298

[42] S. Lee, A. Yang, T.P.II. Moneypenny, J.S. Moore, Kinetically trapped tetrahedral cages via alkyne metathesis. J. Am. Chem. Soc. **138**, 2182–2185 (2016). 10.1021/jacs.6b00468 10.1021/jacs.6b00468 26854552

[43] H. Wang, S. Fang, G. Wu, Y. Lei, Q. Chen et al., Constraining homo- and heteroanion dimers in ultraclose proximity within a self-assembled hexacationic cage. J. Am. Chem. Soc. **142**, 20182–20190 (2020). 10.1021/jacs.0c10253 10.1021/jacs.0c10253 33172262

[44] K. Li, L.Y. Zhang, C. Yan, S.C. Wei, M. Pan et al., Stepwise assembly of Pd_6_(RuL_3_)_8_ nanoscale rhombododecahedral metal-organic cages via metalloligand strategy for guest trapping and protection. J. Am. Chem. Soc. **136**, 4456–4459 (2014). 10.1021/ja410044r 10.1021/ja410044r 24611560

[45] Z. Chang, H. Yang, X. Zhu, P. He, H. Zhou, A stable quasi-solid electrolyte improves the safe operation of highly efficient lithium-metal pouch cells in harsh environments. Nat. Commun. **13**, 1510 (2022). 10.1038/s41467-022-29118-6 10.1038/s41467-022-29118-6 PMC8938510 35314688

[46] Z. Chang, Y. Qiao, H. Yang, X. Cao, X. Zhu et al., Sustainable lithium-metal battery achieved by a safe electrolyte based on recyclable and low-cost molecular sieve. Angew. Chem. Int. Ed. **60**, 15572–15581 (2021). 10.1002/anie.202104124 10.1002/anie.202104124 33884720

