# Supplementary material for: Porous Organic Cage-Based Quasi-Solid-State Electrolyte with Cavity-Induced Anion-Trapping Effect for Long-Life Lithium Metal Batteries
Source: Nanomicro Lett. 2024 Oct 15;17:38. doi: 10.1007/s40820-024-01499-x (PMC11480285; doi:10.1007/s40820-024-01499-x)
Supplement: Supplementary file 1 — Supplementary file1 (DOCX 4301 kb) [file 40820_2024_1499_MOESM1_ESM.docx]

Supporting Information for

**Porous Organic Cage Based Quasi-Solid-State Electrolyte with Cavity-Induced Anion-Trapping Effect for Long-life Lithium Metal Batteries**

Wei-Min Qin^1, #^, Zhongliang Li^2,^ ^#^, Wen-Xia Su^1^, Jia-Min Hu^1^, Hanqin Zou^1^, Zhixuan Wu^1^, Zhiqin Ruan^1^, Yue-Peng Cai^1,3,*^, Kang Li^1,3,*^, and Qifeng Zheng^1,3,*^

^1^School of Chemistry, South China Normal University, Guangzhou 510006, P. R. China

^2^Key Laboratory of Functional Metal-Organic Compounds of Hunan Province, College of Chemistry and Material Science, Hengyang Normal University, Hengyang 421008, P. R. China

^3^Guangzhou Key Laboratory of Energy Conversion and Energy Storage Materials, Guangzhou 510006, P. R. China

^#^Wei-Min Qin and Zhongliang Li contributed equally to this work

*Corresponding authors. E-mail: [caiyp@scnu.edu.cn](mailto:caiyp@scnu.edu.cn) (Yue-Peng Cai); [likang5@m.scnu.edu.cn](mailto:likang5@m.scnu.edu.cn) (Kang Li); [qifeng.zheng@m.scnu.edu.cn](mailto:qifeng.zheng@m.scnu.edu.cn) (Qifeng Zheng)

**S1 Experimental Procedures**

***Materials and solvents:*** Polyvinylidene difluoride (PVDF, Mw: 1,000,000 g mol^−1^ ) was purchased from ARKEMA and dried under vacuum at 60 °C for 24 h before use. Bis(trifluoromethane)sulfonamide lithium salt (LiTFSI) and Propylene carbonate (PC) was kindly provided by Guangzhou Tinci Materials Technology Co., Ltd. 1,3,5-Triformylbenzene (96%) and (R,R)-1,2-diaminocyclohexane (98%) was purchased from Bide Pharmatech Co., Ltd. Polytetrafluoroethylene (PTFE) emulsion (60wt.%) was purchased from Guangdong Canrd New Energy Technology Co., Ltd. Trifluoroacetic acid (98%) was purchased from Energy Chemical Co., Ltd. Dichloromethane (99%) and Methanol (99%) was purchased from Tianjin Zhiyuan Chemical Reagent Co., Ltd.

***Material characterization:*** X-ray powder diffraction (XRD) patterns were collected using a diffractometer (D8 Advance, Bruker) with Cu K𝛼 radiation. The morphologies were observed using a scanning electron microscope (MAIA3, TESCAN). N_2_ adsorption/desorption isothermal test was recorded on a Micromeritics (Belsorp-max, MicrotracBel). Thermogravimetric analysis (TGA) was tested from 30 to 900°C at 10 °C min^−1^ using a thermogravimetric analyzer (209 F3, Netzsch). The nuclear magnetic resonance (NMR) analysis of the materials was carried out on spectrometer (Avance NEO 600 MHz, Bruker). The chemical composition on the surface of electrode was analyzed by XPS (PHI 1600 ESCA, PerkinElmer). The Raman spectrum of the electrolyte was obtained from a spectrometer (inVia, Renishaw) with an emission wavelength of 785 nm. The content of the LiTFSI in QSSE was estimated using an inductively coupled plasma optical emission spectrometry (ICP-OES, SPECTRO ARCOS MV)

***Electrochemical measurement:*** The ionic conductivities of the QSSE were determined using electrochemical impedance spectroscopy (EIS) measurement by assembling the QSSE between two stainless-steel (SS) blocking electrodes in a CR2032 cell configuration. The measurements were conducted at a temperature ranging from 303 to 363 K with 10 mV of AC amplitude within the frequency ranging from 10^6^ to 0.1 Hz using a Potentiostat (VMP3, Bio-Logic). The ionic conductivity (σ, S cm^−1^ ) of QSSE was calculated by Eq. S1.

$\sigma=\frac{R}{L\times S}$ (S1)

where$L$ (cm) is the thickness of QSSE, $R$ (Ω) is the impedance obtained from the real axis in the Nyquist plot, and $S$ (cm^−2^ ) is the area of QSSE.

The active energy (Ea) of ionic transport was calculated by Eq. S2.

$\sigma=Ae^{\frac{-E_{a}}{RT}}$ (S2)

where $A$ is the pre-exponential factor, $T$ is the testing temperature, $R$ is gas constant.

The Li^+^ transference number (t_Li_^+^) of QSSE was determined by the AC impedance and direct-current (DC) polarization using a symmetric Li||Li cell at 25 °C. A DC polarization voltage ($\Delta V$) of 10 mV was applied for 7200 s to obtain the steady current. The$t_{{Li}^{+}}$ was calculated by Eq. S3.

$t_{{Li}^{+}}=\frac{I_{s}\times(\Delta V-I_{0}R_{0})}{I_{0}\times(\Delta V-I_{s}R_{s})}$ (S3)

$I_{0}$ and $R_{0}$ are the initial current (mA) and charge-transfer resistance (Ω) before polarization, respectively. $I_{s}$ and $R_{s}$ are the steady state current and charge-transfer resistance after polarization, respectively.

The electrochemical stability window of the QSSE was tested by linear sweep voltammetry (LSV) from open circuit voltage (OCV) to 6.0 V of a Li||SS cell at a sweep rate of 1.0 mV s^−1^. The charge-discharge tests of the Li||Li symmetrical and Li||LFP cells were performed by a LAND tester (CT2001A, Wuhan Jinnuo Electronic) at different current densities. The Li||LFP cells were tested with voltage range of 2.8-3.9 V. The critical current densities (CCD) was measured by testing Li||Li symmetric cells under the initial current density of 0.05 mA cm^−2^ with an increasing step of 0.05 mA cm^−2^ per cycle.

***Theoretical Calculation:*** The adsorption energy calculations of the anion and molecule cage complex were performed using the Gaussian 16 package. The structures of the molecule cage and anion were optimized at the B3LYP/6-311G(d) level for C, H, O, F, N, and S elements. Frequency calculations were performed at the same level to confirm the obtained optimized stationary point [S 1,2].The adsorption energy ($E_{A}$) between TFSI^−^ anion and POC (CC3 or TD_A_) is defined as Eq. S4:

$E_{A}=E_{complex}-E_{POC}-E_{anion}$ (S4)

where $E_{complex}$ is the total energy of molecule cage-anion complex, $E_{POC}$ is the energy of a free POC unit, $E_{anion}$ is the energy of an anion.

**S2 Supplementary Figures and Tables**


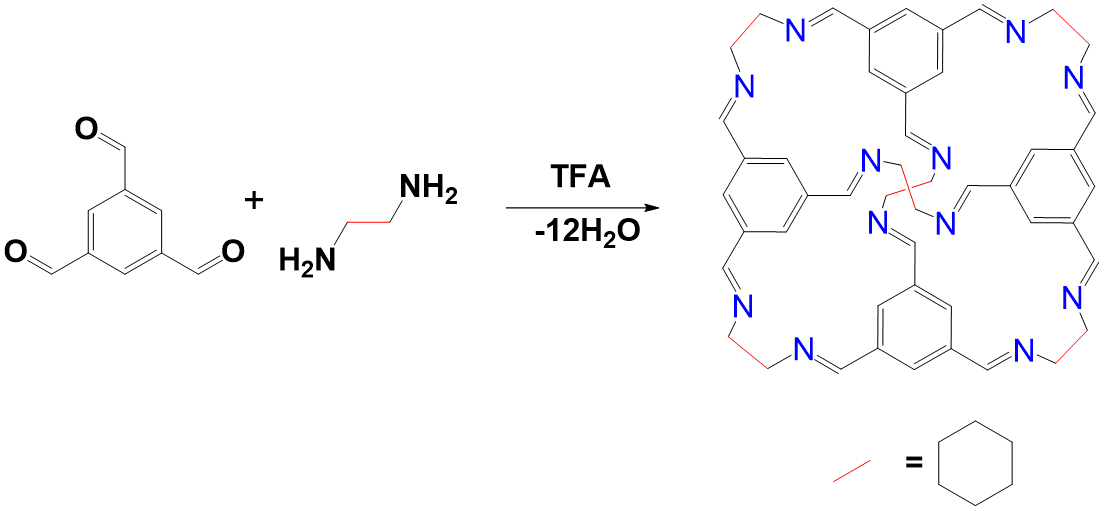


**Fig. S1** Synthesis of CC3


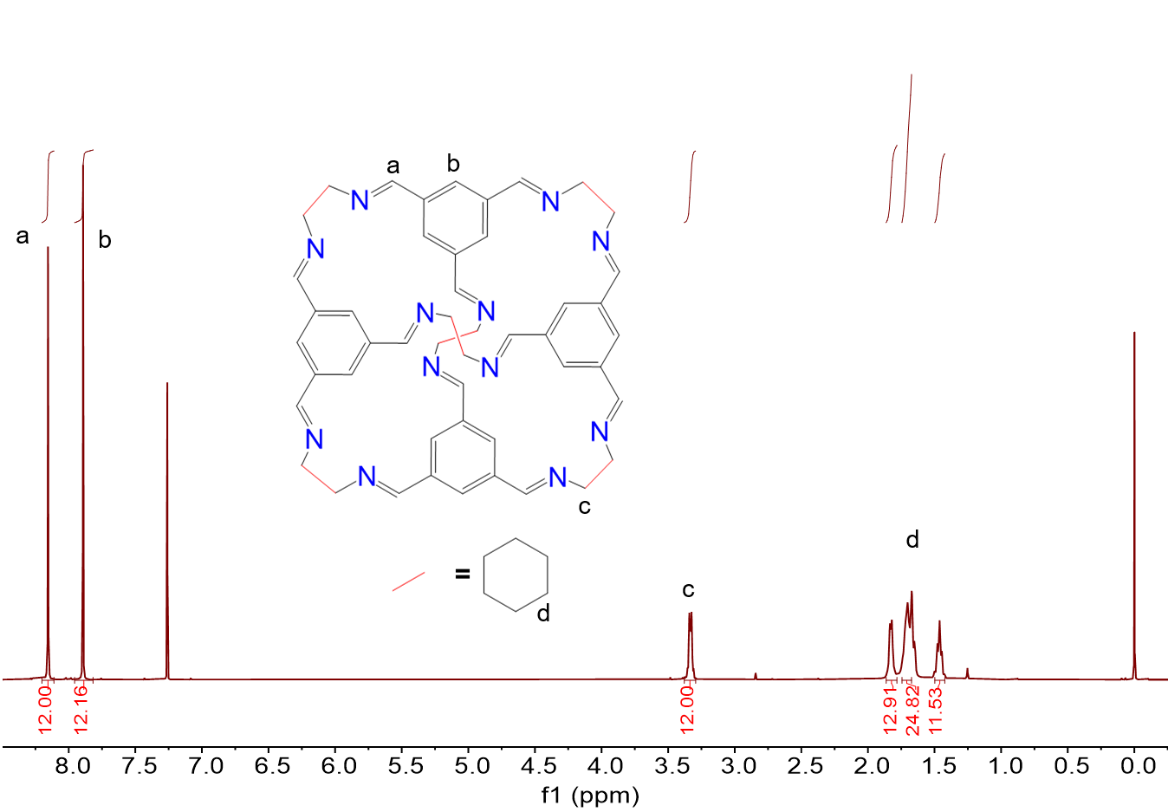


**Fig. S2** ^1^H NMR of CC3 in CDCl_3_


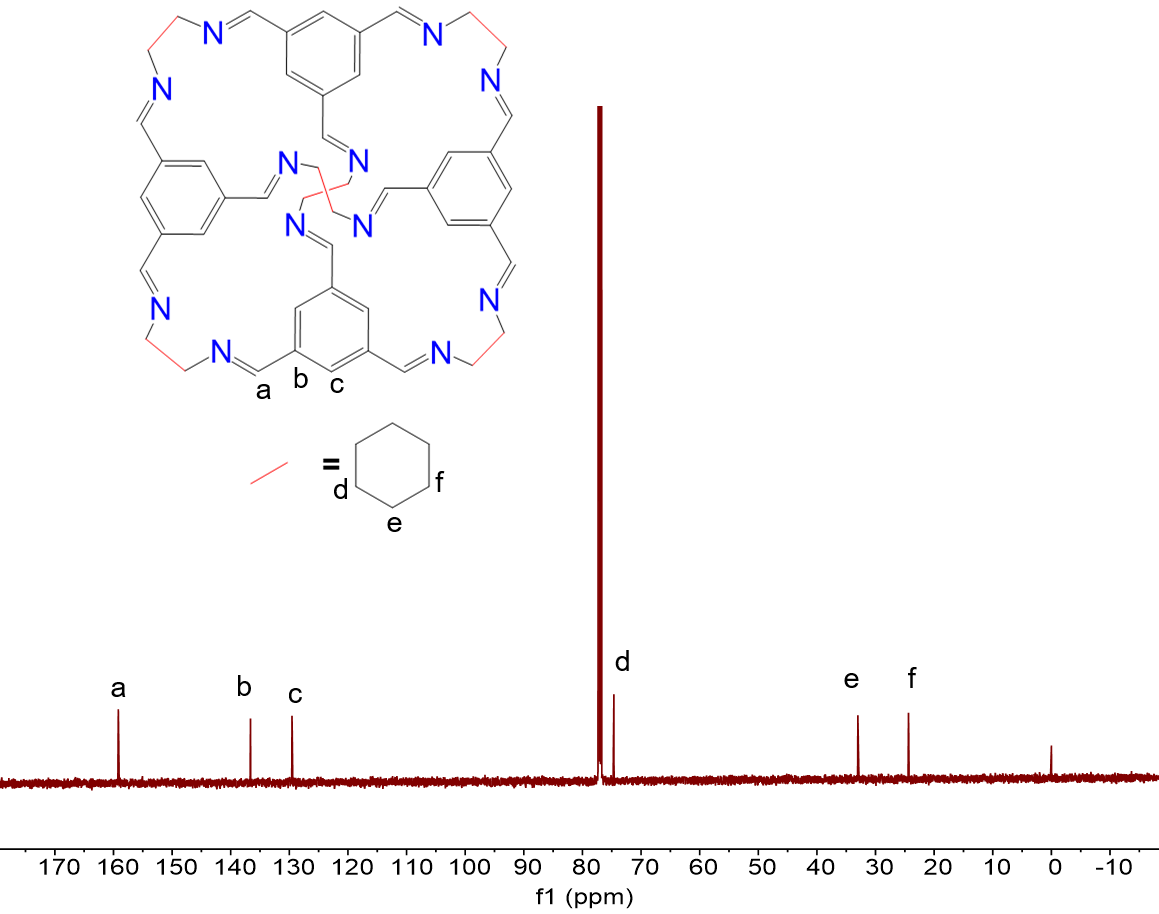


**Fig. S3** ^13^C NMR of CC3 in CDCl_3_


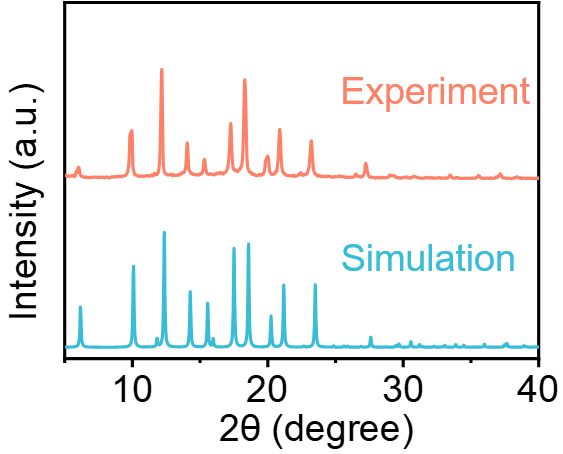


**Fig. S4** X-ray diffraction patterns of CC3


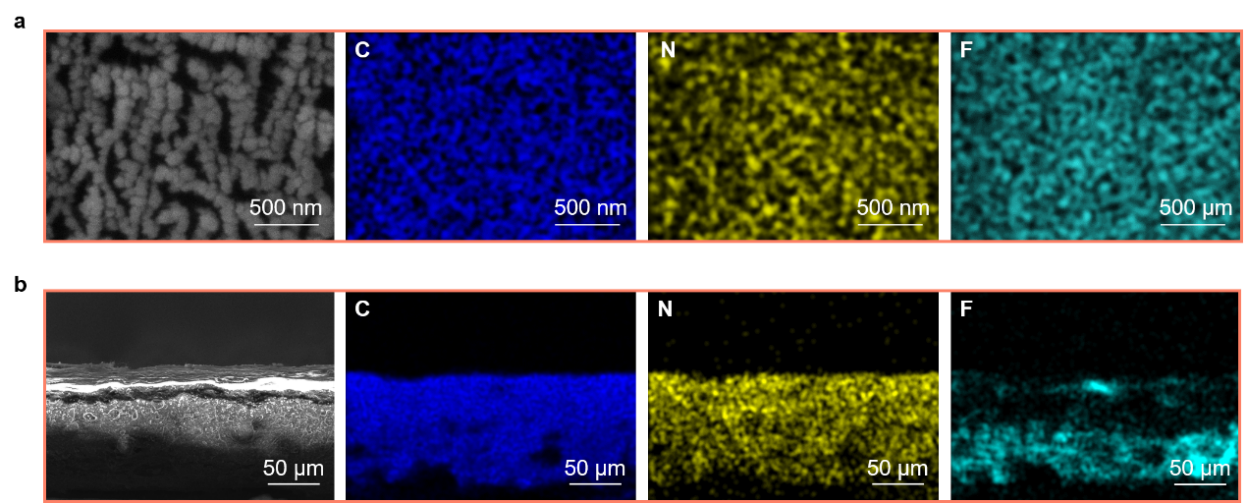


**Fig. S5** SEM images and corresponding EDS element mapping images of the CC3/PTFE film


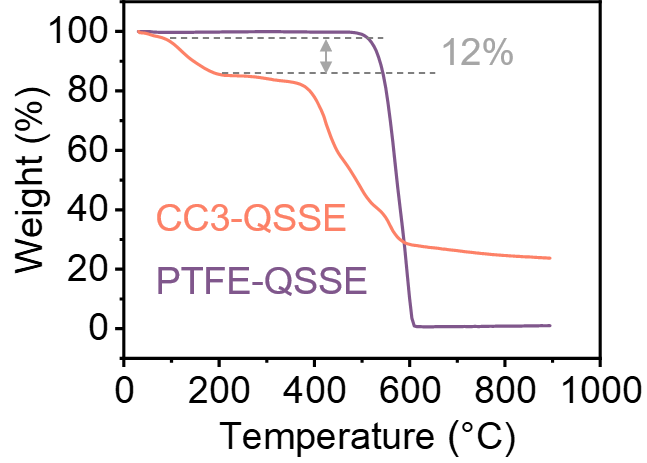


**Fig. S6** TGA curves of CC3-QSSE and PTFE film soaked in the liquid electrolyte


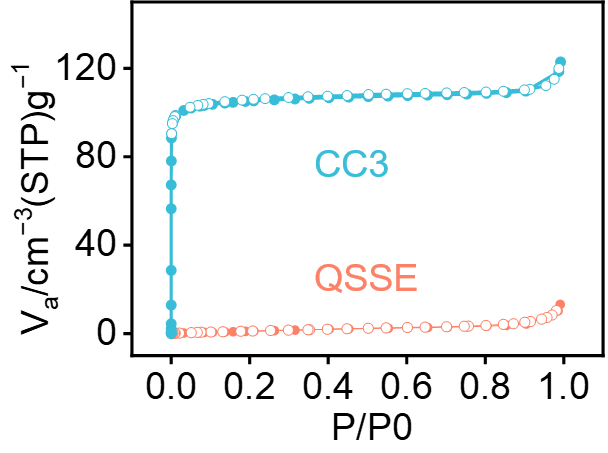


**Fig. S7** Nitrogen adsorption and desorption curves of CC3 and QSSE

**
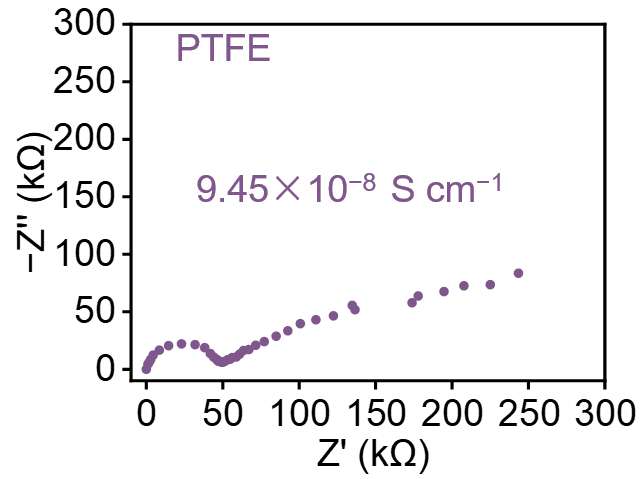
**

**Fig.** **S8** Ionic conductivity of pure PTFE film soaked with liquid electrolyte at 25 °C

**
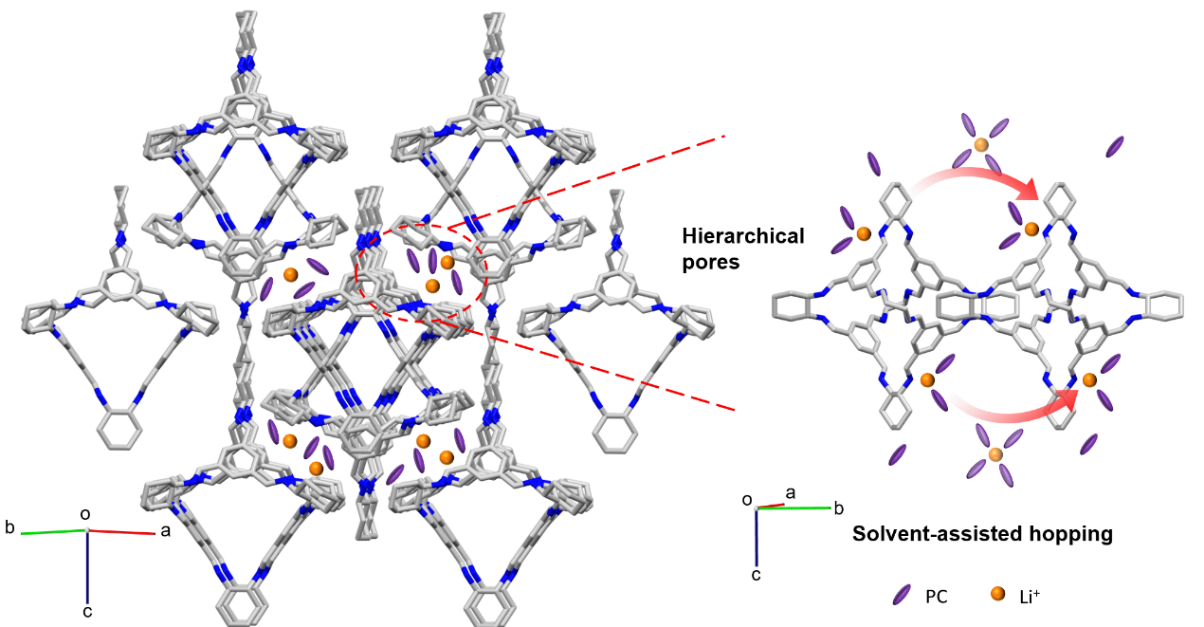
**

**Fig. S9** Schematic illustration of ion transport mechanism in CC3-based QSSE. The hierarchical pores formed by CC3 stacking serve as Li^+^ transport channels, and Li^+^ are transported through solvent-assisted hopping mechanism


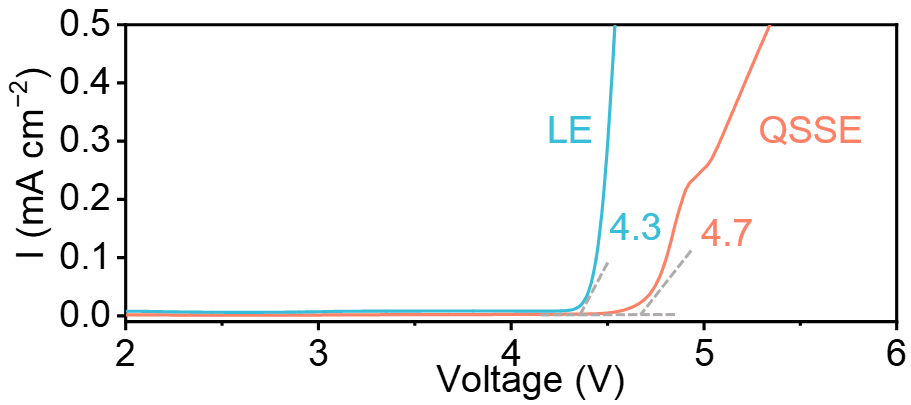


**Fig. S10** LSV of QSSE and LE


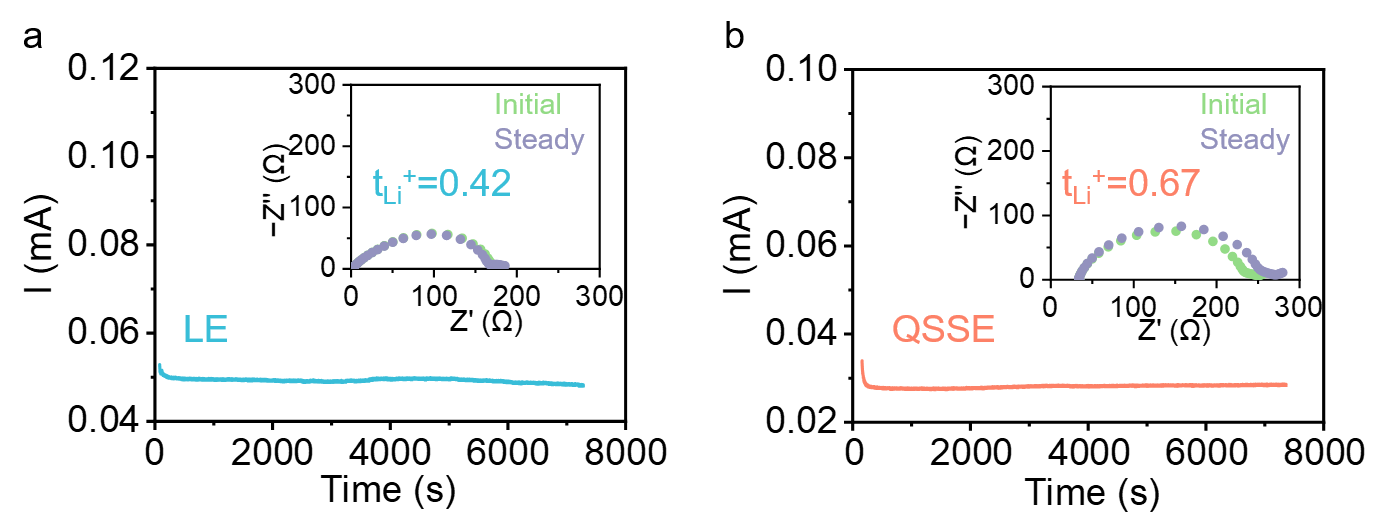


**Fig. S11** Transference number measurement of (**a**) LE and (**b**) QSSE


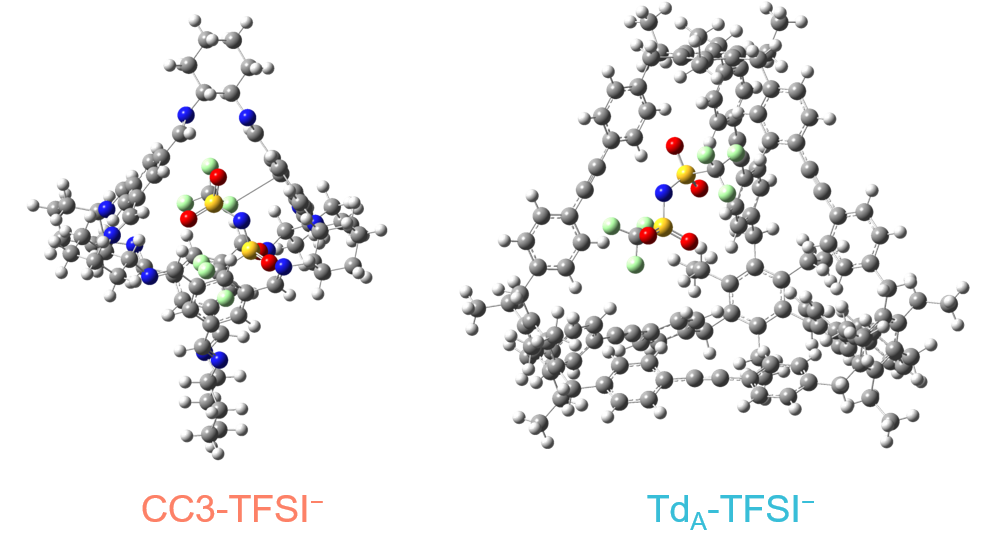


**Fig. S12** Optimized structure of TFSI^−^ in the cavity of CC3 and Td_A_


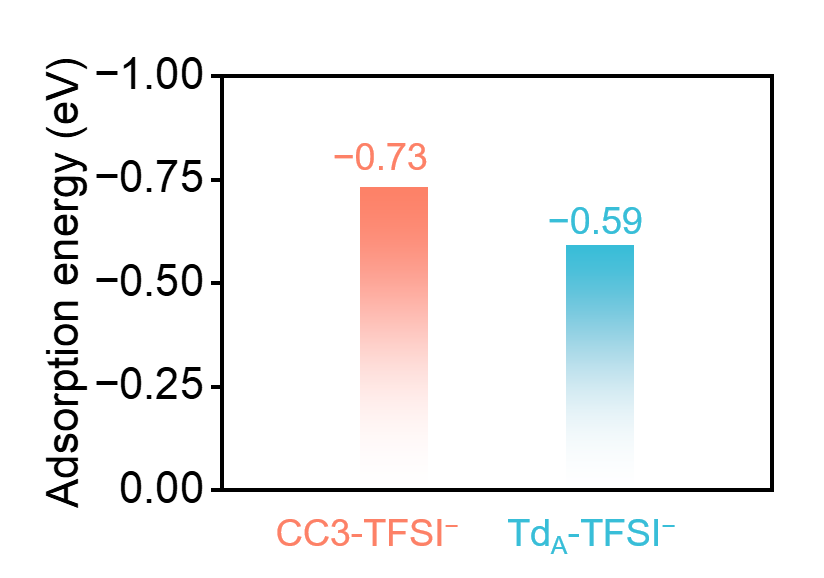


**Fig. S13** The adsorption energy of TFSI^−^ in the cavity of CC3 and Td_A_


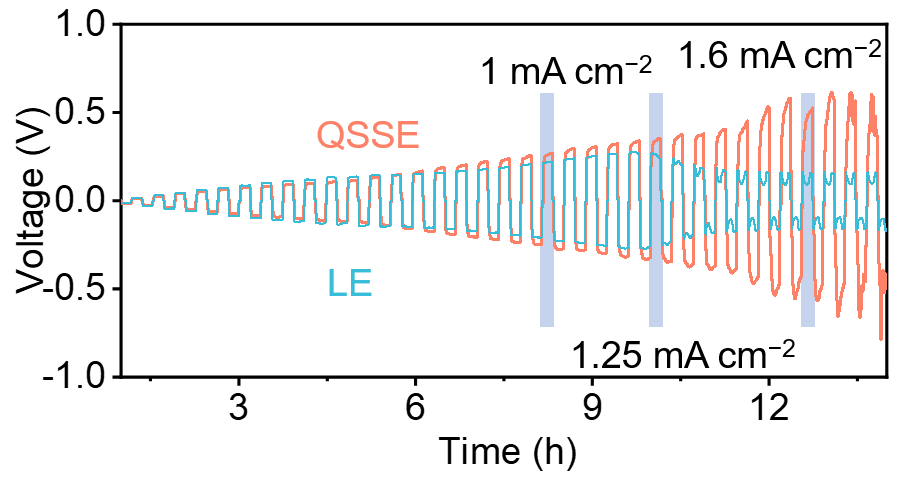


**Fig. S14** Critical current density(CCD) measurement of Li||Li symmetric cell using QSSE and LE


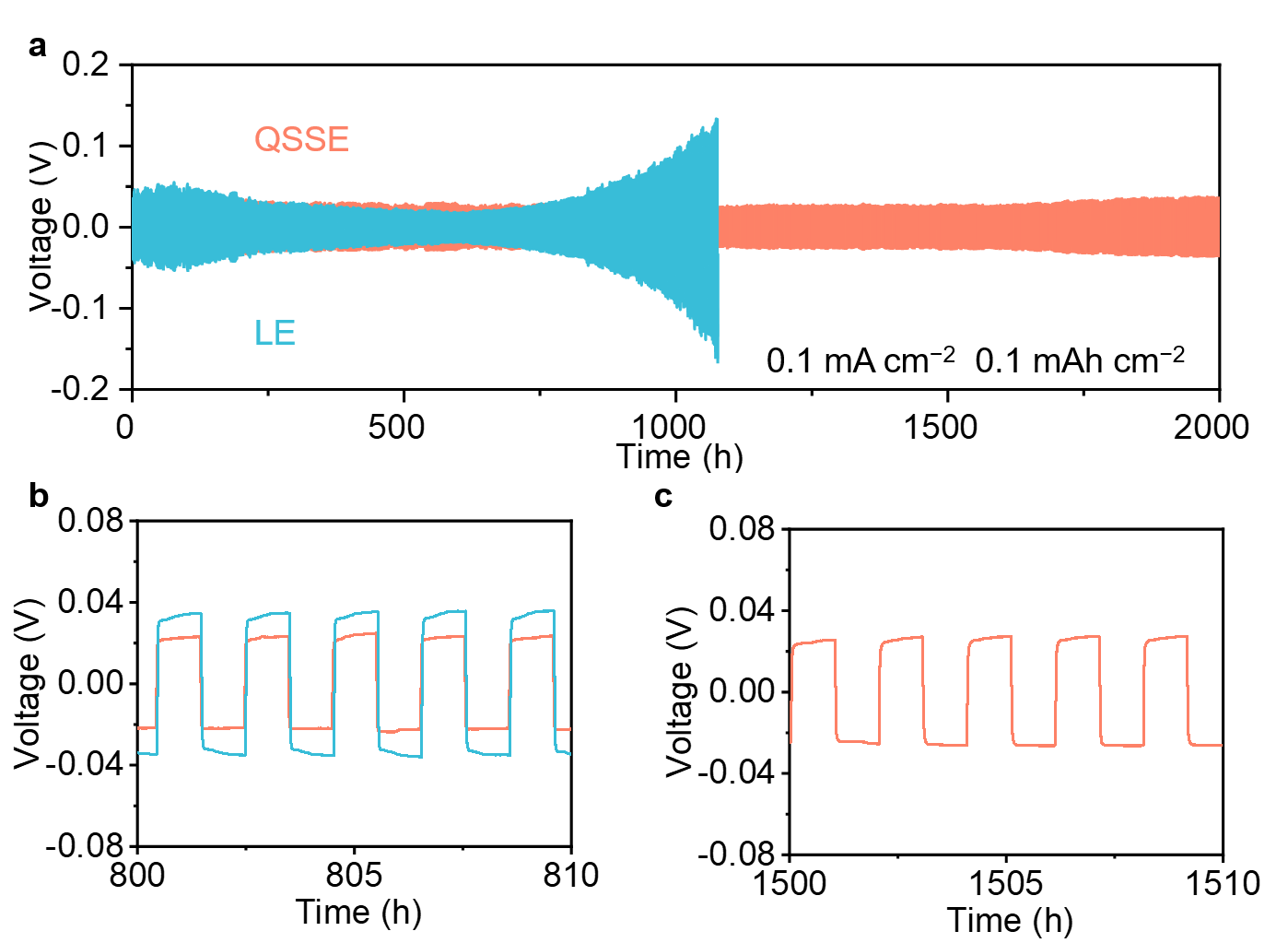


**Fig. S15** (**a**) Long-term cycling of Li||Li symmetric cells at 0.1 mA cm^−2^ for 0.1 mAh cm^−2^. (**b**) and (**c**) Enlarged voltage profiles at different stages


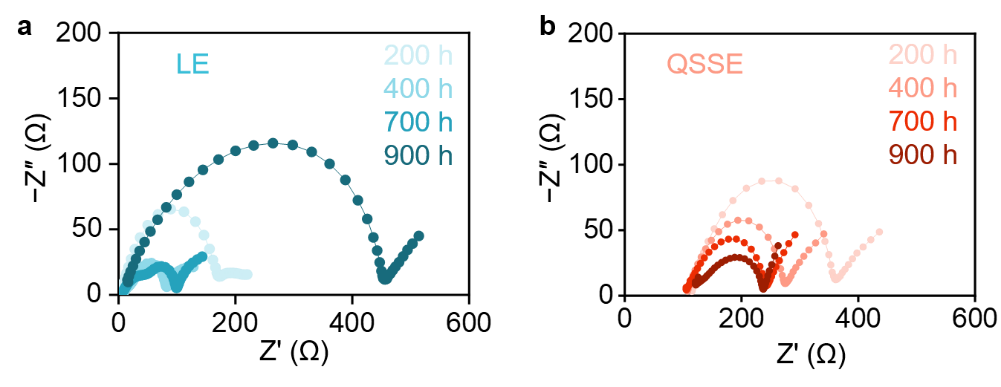


**Fig. S16** EIS spectra of Li||Li symmetric cells using (**a**) LE and (**b**) QSSE at different plating/stripping time at 0.1 mA cm^−2^ for 0.1 mAh cm^−2^


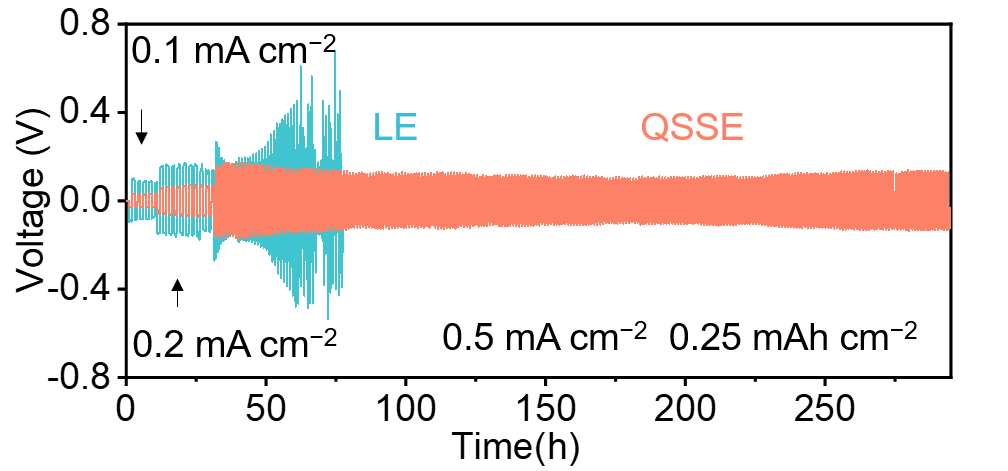


**Fig. S17** Long-term cycling of Li||Li symmetric cells at 0.5 mA cm^−2^ for 0.25 mAh cm^−2^


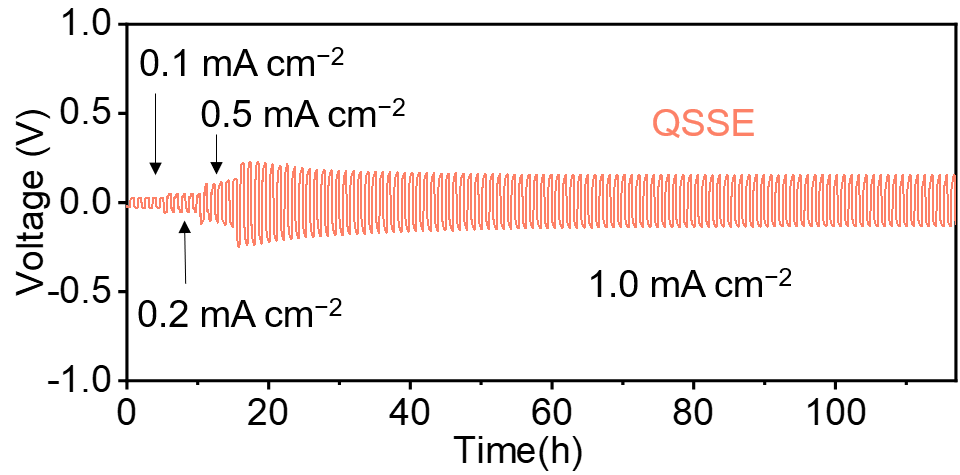


**Fig.** **S18** Long-term cycling of Li||Li symmetric cell at 1.0 mA cm^−2^ for 0.5 mAh cm^−2^


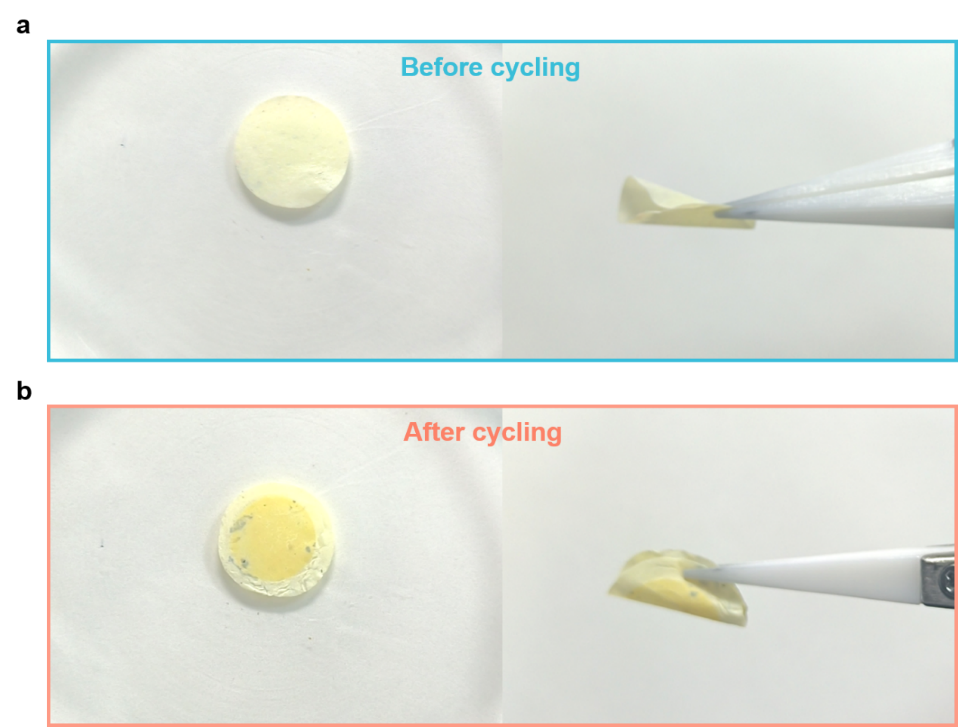


**Fig. S19** The photographs of QSSE film in Li||Li symmetric cells (**a**) before cycling and (**b**) after cycling


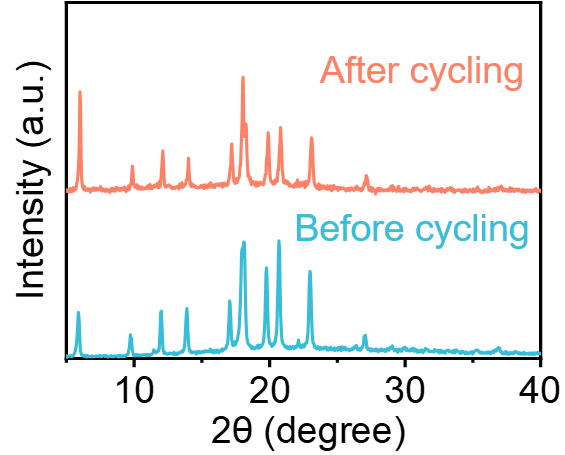


**Fig. S20** X-ray diffraction patterns of QSSE before and after cycling in Li||Li symmetric cells


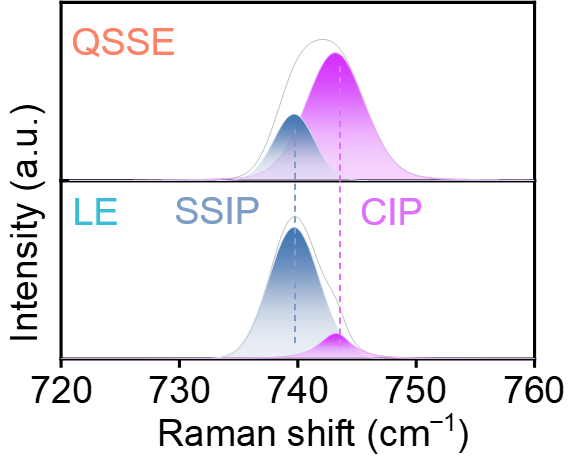


**Fig. S21** Raman spectra of LE and QSSE


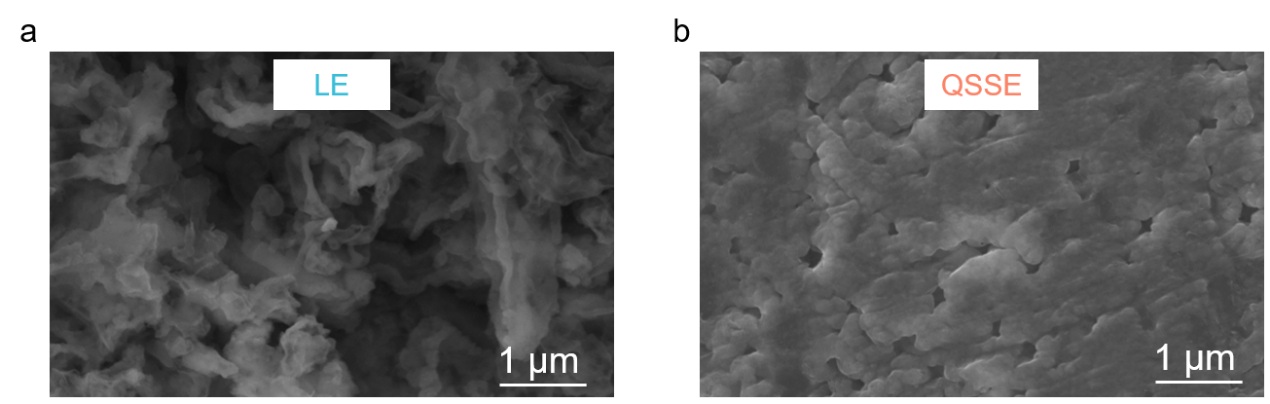


**Fig. S22** SEM images of Li-metal anode after 1000 cycles in Li||LFP cells with (**a**) LE and (**b**) QSSE

**
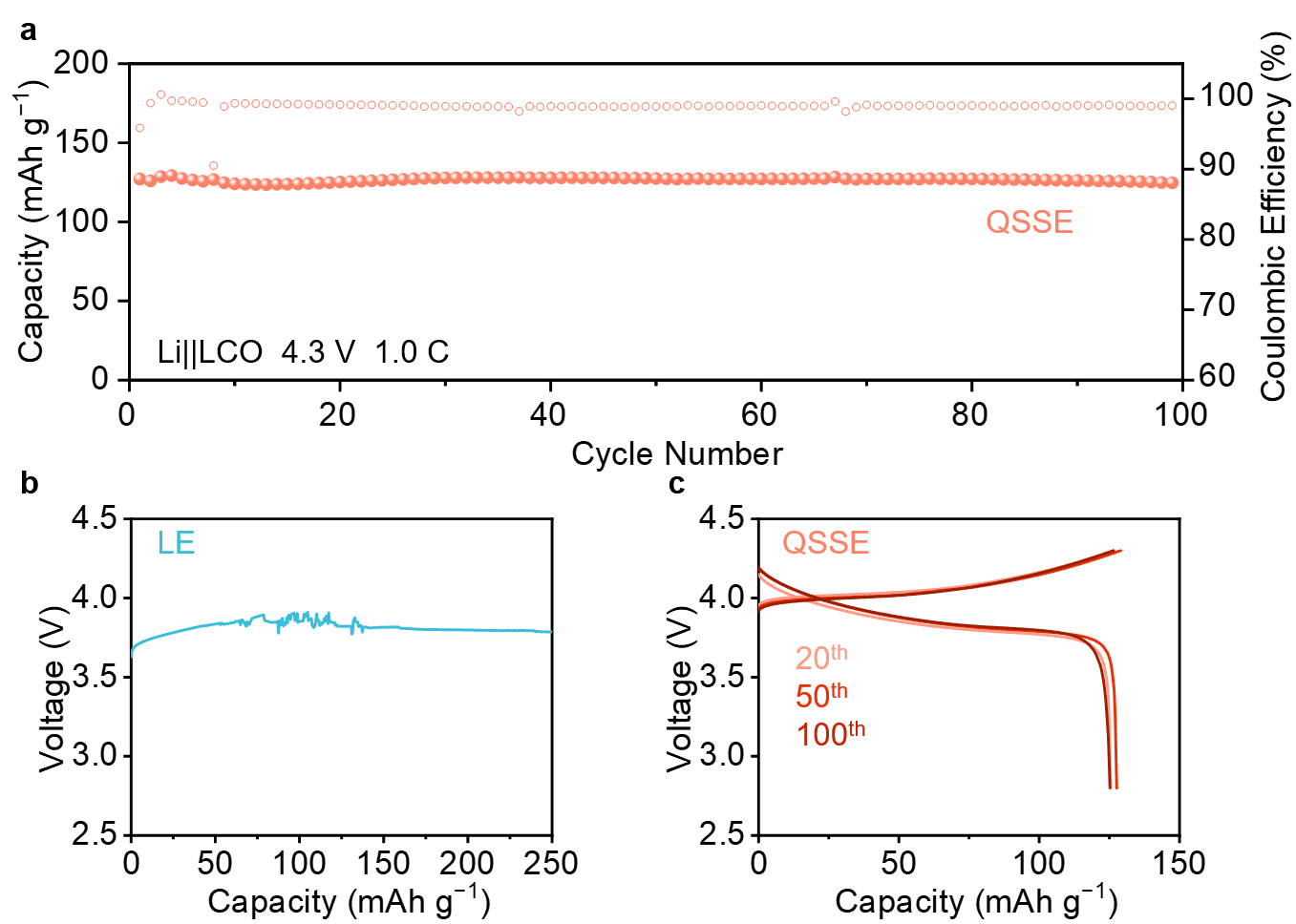
**

**Fig. S23** Electrochemical performance of Li||LCO cells at room temperature. (**a**) Long-term cycling of Li||LCO cells using QSSE with a cut-off voltage of 4.3 V at 1.0 C. Charge-discharge curves for Li||LCO cells using (**b**) LE and (c) QSSE

**Table S1** Electrochemical performances comparison of this work with reported works

| **QSSE** | **σ (S cm^−1^)** | **AC** | **Cathode**  **material** | **Cycling**  **condition** | **Capacity retention** | **Refs.** |
| --- | --- | --- | --- | --- | --- | --- |
| LiOOC‐COF3 | 1.36×10^−5^ (30 ^o^C) | LiPF_6_ in EC/DEC | C_6_O_6_ | 600 cycles, 50 mA g^−1^, 25 °C,  4.0 V | ~90% | [S3] |
| COF-SS-Li | 1.28×10^−4^ (30 ^o^C) | LiPF6 EC/DMC | LiFePO_4_ | 500 cycles, 100 mA g^−1^ _,_ 25 °C | 57% | [S4] |
| SE-4Cl-Li | 2.16×10^−4^  (25 ^o^C) | LiTFSI in PC | LiFePO_4_ | 1500 cycles,  1 C, 25 °C | 90% | [S5] |
|  |  |  | NCM811 | 100 cycles, 1 C, 25 °C, 4.3 V | 82% |  |
| HKUST-1 QSE | 1.02×10^−3^  (30 ^o^C) | LiClO_4_ in PC | LiFePO_4_ | 210 cycles,  1 C, 30 °C | 93% | [S6] |
|  |  |  | NCM811 | 50 cycles,  0.2 C 30 °C,4.3V | ~70% |  |
| PEO/ZIF  -90-g-IL | 1.17×10^−4^ (30 ^o^C) | ILs | LiFePO_4_ | 500 cycles,  2 C 60 °C | 71% | [S7] |
|  |  |  | NCM811 | 100 cycles, 0.1 C, 60 °C, 4.2 V | 63% |  |
| Si@LATP  /PVDF/PVC | 1.06×10^−3^ (25 ^o^C) | FEC/TEP | LiFePO_4_ | 200 cycles,  0.5 C 25 °C | ~100% | [S8] |
|  |  |  | NCM811 | 100 cycles, 0.5 C, 25 °C, 4.2 V | 86% |  |
| MOF@IL | 4.08×10^−4^ (30 ^o^C) | LiTFSI in ILs | LiFePO_4_ | 500 cycles,  0.5 C, 30 °C | 96% | [S9] |
|  |  |  | NCM811 | 100 cycles, 1 C, 30 °C, 4.3 V | ~82% |  |
| PILH | 1.91×10^−4^ (30 ^o^C) | LiTFSI in ILs | LiFePO_4_ | 150 cycles,  0.5 C, 30 °C | 95% | [S10] |
|  |  |  | NCM523 | 50 cycles, 0.2 C, 60 °C, 4.3 V | 72% |  |
| Li@Zn-MOF-74/Li-IL | 1.73×10^−4^ (30 ^o^C) | LiTFSI in ILs | LiFePO_4_ | 500 cycles,  0.5C, 30 °C | ~90% | [S11] |
|  |  |  | LiCoO_2_ | 100 cycles, 0.1C, 30 °C, 4.2 V | ~70% |  |
| SPE2-PI-ZIF8 | 4.7×10^−4^ (25 ^o^C) | LiPF_6_ in EC/DEC/DMC | LiFePO_4_ | 300 cycles,  1 C, 25 °C | ~80% | [S12] |
|  |  |  | NCM523 | 100 cycles, 0.5 C, 25 °C, 4.3 V | 96 % |  |
| Li-PEG@NUST-23 | 1.17×10^−4^ (10 ^o^C) | LiTFSI in PEG | LiFePO_4_ | 84 cycles  0.1 C, 10 °C | ~100% | [S13] |
| PMLSE | 2.0×10^−4^ (25 ^o^C) | LiTFSI in EC: DEC | LiFePO_4_ | 200 cycles  1 C, 25 °C | 86% | [S14] |
|  |  |  | LiCoO_2_ | 200 cycles, 0.2 C, 25 °C, 4.2 V | 87% |  |
| **CC3-QSSE** | **1.25×10^−4^ (25 ^o^C)** | **LiTFSI in PC** | **LiFePO_4_** | **1000 cycles,**  **0.5 C, 25 °C** | **85%** | **This**  **work** |
|  |  |  | **LiCoO_2_** | **100 cycles, 1 C, 25 °C, 4.3 V** | **98%** |  |

Abbreviations: AC = additional components.

**Supplementary References**

[S1] A. V. Marenich, C. J. Cramer, D. G. Truhlar, Performance of SM6, SM8, and SMD on the SAMPL1 test set for the prediction of small-molecule solvation free energies. J. Phys. Chem. B **113**, 4538-4543 (2009). <http://doi:10.1021/jp809094y>

[S2] J. Yang, Y. Zhang, Z. Li, X. Xu, X. Su et al., Three birds with one stone: tetramethylurea as electrolyte additive for highly reversible Zn-metal anode. Adv. Funct. Mater. **32**, 2209642 (2022). <http://doi:10.1002/adfm.202209642>

[S3] G. Zhao, Z. Mei, L. Duan, Q. An, Y. Yang et al., COF-based single Li^+^ solid electrolyte accelerates the ion diffusion and restrains dendrite growth in quasi-solid-state organic batteries. Carbon Energy **5**, e248 (2022). <http://doi:10.1002/cey2.248>

[S4] J. Zhang, D. Luo, H. Xiao, H. Zhao, B. Ding et al., Post-synthetic covalent organic framework to improve the performance of solid-state Li^+^ electrolytes. ACS Appl. Mater. Interfaces **15**, 34704-34710 (2023). <http://doi:10.1021/acsami.3c03643>

[S5] W. He, D. Li, S. Guo, Y. Xiao, W. Gong et al., Redistribution of electronic density in channels of metal-organic frameworks for high-performance quasi-solid lithium metal batteries. Energy Storage Mater. **47**, 271-278 (2022). <http://doi:10.1016/j.ensm.2022.02.003>

[S6] H. Liu, H. Pan, M. Yan, X. Zhang, Y. Jiang, Extraordinary ionic conductivity excited by hierarchical ion-transport pathways in MOF-based quasi-solid electrolytes. Adv. Mater. **35**, 2300888 (2023). <http://doi:10.1002/adma.202300888>

[S7] Z. Lei, J. Shen, J. Wang, Q. Qiu, G. Zhang et al., Composite polymer electrolytes with uniform distribution of ionic liquid-grafted ZIF-90 nanofillers for high-performance solid-state Li batteries. Chem. Eng. J. **412**, 128733 (2021). <http://doi:10.1016/j.cej.2021.128733>

[S8] Y. Jin, X. Zong, X. Zhang, Z. Jia, H. Xie et al., Constructing 3D Li^+^-percolated transport network in composite polymer electrolytes for rechargeable quasi-solid-state lithium batteries. Energy Storage Mater. **49**, 433-444 (2022). <http://doi:10.1016/j.ensm.2022.04.035>

[S9] L. Du, B. Zhang, W. Deng, Y. Cheng, L. Xu et al., Hierarchically self-assembled MOF network enables continuous ion transport and high mechanical strength. Adv. Energy Mater. **12**, 2200501 (2022). <http://doi:10.1002/aenm.202200501>

[S10] Z. Zhang, Y. Huang, H. Gao, C. Li, J. Hang et al., MOF-derived multifunctional filler reinforced polymer electrolyte for solid-state lithium batteries. J. Energy Chem. **60**, 259-271 (2021). <http://doi:10.1016/j.jechem.2021.01.013>

[S11] P. Dong, X. Zhang, W. Hiscox, J. Liu, J. Zamora et al., Toward high-performance metal-organic-framework-based quasi-solid-state electrolytes: tunable structures and electrochemical properties. Adv. Mater. **35**, 2211841 (2023). <http://doi:10.1002/adma.202211841>

[S12] G. Wang, P. He, L. Z. Fan, Asymmetric polymer electrolyte constructed by metal-organic framework for solid-state, dendrite-free lithium metal battery. Adv. Funct. Mater. **31**, 2007198 (2020). <http://doi:10.1002/adfm.202007198>

[S13] Y. Xuan, Y. Wang, B. He, S. Bian, J. Liu et al., Covalent organic framework-derived quasi-solid electrolyte for low-temperature lithium-ion battery. Chem. Mater. **34**, 9104-9110 (2022). <http://doi:10.1021/acs.chemmater.2c01982>

[S14] J. Sun, X. Yao, Y. Li, Q. Zhang, C. Hou et al., Facilitating interfacial stability via bilayer heterostructure solid electrolyte toward high-energy, safe and adaptable lithium batteries. Adv. Energy Mater. **10**, 2000709 (2020). <http://doi:10.1002/aenm.202000709>
